# Machine Learning-Based Predictive Modeling of Infrared Spectroscopic Data from Thermal Conversion of Athabasca Bitumen

**DOI:** 10.1021/acsomega.5c04463

**Published:** 2025-07-02

**Authors:** Noora Al Mansoori, Munawar Abdul Shaik, Kaushik Sivaramakrishnan

**Affiliations:** Department of Chemical and Petroleum Engineering, 105964 UAE University, Khalifa Street, Al Ain 15551, United Arab Emirates (UAE)

## Abstract

This study explores the use of machine learning (ML) techniques to predict Fourier-transform infrared (FTIR) intensities of products from the thermal cracking of Athabasca bitumen, aiming to develop a reliable soft-sensor. The ultimate goal is to obtain the FTIR spectra of the thermally cracked products online to reduce process time from slow physical measurements. Various ML models, including Linear Regression (LinR), partial least squares regression (PLSR), support vector regression (SVR), K-nearest neighbors (*k*-NN), random forest (RF), and gradient boosting regression (GBR), were implemented to enhance the predictive accuracy and efficiency of FTIR spectroscopy, aiming to reduce the need for traditional physical measurements which are often slow compared to the rapid predictions offered by ML techniques. To assess the model’s generalization capabilities, with respect to model predictions, the models were trained and tested across four different scenarios with varying temperature data obtained from visbreaking experiments performed on Athabasca Bitumen at temperatures ranging from 25 to 420 °C with reaction times ranging from 15 min to 27 h. Scenario 1 included all 61,740 data points utilizing an 80/20 train-test split with 10-fold cross-validation (CV). Scenario 2 involved training on temperatures of 25, 350, and 400 °C and testing on 300, 380, and 420 °C. Scenario 3 involved training on temperatures of 350, 380, and 400 °C and testing on 25, 300, and 420 °C. Finally, Scenario 4 involved training on temperatures of 25, 300, 350, and 380 °C and testing on 400 and 420 °C. Bayesian optimization was employed for hyperparameter tuning to identify the optimal configurations for each model. The results indicate that ensemble methods, particularly GBR, consistently achieved the highest predictive accuracy (*R* ^2^) and lowest root mean squared error (RMSE) across all scenarios. In Scenario 1, GBR achieved a prediction accuracy of 99.66%. Scenario 2 highlighted the models’ ability to generalize across varying temperatures, with both RF and GBR achieving similar performance with high prediction accuracies of around 94%. Scenario 3, characterized by significant temperature variability, demonstrated the robustness of GBR, which outperformed RF and *k*-NN with a predictive accuracy of 92.15%. Scenario 4, focusing on high-temperature predictions from low-temperature training data, showed that GBR still performed robustly with a predictive accuracy of 80.40%. The study concludes that GBR models, particularly those with well-tuned hyperparameters, are highly effective in predicting FTIR intensities, outperforming other techniques like RF, *k*-NN, LinR, and PLSR. The integration of advanced ML techniques and Bayesian optimization significantly enhances the capability to predict FTIR spectra, providing a reliable soft-sensor as an alternative to traditional physical experimentation methods. This approach not only saves time and resources but also ensures consistent and high-quality predictive performance in chemical analysis and monitoring.

## Graphical abstract

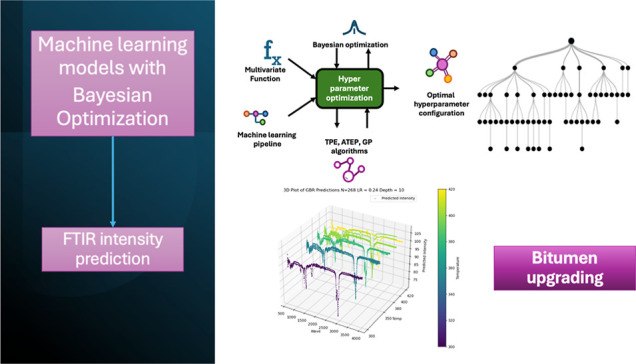

## 1 Introduction

Machine Learning (ML) techniques are revolutionizing process systems engineering and chemical reaction engineering by enabling accurate predictions of complex multidimensional data from traditional analytical characterization methods, including Fourier-transform infrared (FTIR) spectroscopy, nuclear magnetic resonance (NMR), electron spin resonance (ESR) spectroscopy, thermogravimetric analysis (TGA), and gas chromatography paired with advanced detectors. These characterization techniques utilize sophisticated and advanced experimental tools, but the disadvantage is that the measurements are time-consuming, resource intensive and slows down the overall chemical process. The characterizations are done on both the feedstock and the products, but in this work, the focus is on reproducing the FTIR data from products of visbreaking (mild thermal cracking) experiments of Athabasca bitumen. FTIR combined with attenuated total reflectance (ATR) is a critical tool in the analysis of various functional groups present in the product samples in order to determine the reaction chemistry involved in the thermal conversion of complex feedstocks such as bitumen.^1–4^ The integration of data-driven ML models as soft sensors offers a promising alternative by enabling the prediction of FTIR spectra through the utilization of historical data without the need for continuous physical experimentation. Once modeled and tuned, ML approaches are highly flexible, robust, versatile, and 3–4 order of magnitudes quicker than offline instruments in obtaining the characterization data. This also reduces plausible human errors arising from sample preparation and data collection.

Athabasca bitumen, derived from the Athabasca Oil Sands in Alberta, Canada, is a major natural resource notable for its unique characteristics and its complex molecular structure.^5^ It is a raw, highly viscous form of petroleum embedded in sand and clay.^6^ Noncatalytic thermal cracking of bitumen at low temperature (visbreaking), breaks down the large, complex hydrocarbon molecules into smaller, lighter molecules, significantly reducing the viscosity and enabling it to flow more freely. Jaramillo et al.^7^ studied the thermal conversion of Cold Lake bitumen at 150–300 °C, demonstrating that viscosity decreases significantly at higher temperatures (250–300 °C) due to a shift from free-radical addition to cracking reactions. The main findings concluded that mild thermal conversion successfully reduces viscosity while preserving liquid yield and minimizing olefin content, offering a cost-efficient method for transporting heavy crude oil. Similarly, Sivaramakrishnan et al.^1^ conducted thermal conversion experiments on Athabasca bitumen at 300–420 °C, investigating two approaches: one without solvent extraction, where viscosity reduction was achieved but influenced by coke formation, and another with solvent extraction, which further enhanced viscosity reduction by separating liquid products from insoluble solids. It was concluded that postreaction procedures, such as solvent selection and rheological conditions during viscosity measurements, significantly impact the observed viscosity and the characterization of thermally converted products. While both studies provide essential insights into viscosity reduction mechanisms, they differ in feedstock, temperature ranges, and methodologies. In fact, the data set generated by Sivaramakrishnan et al.^1^ serves as the foundation of the present study, which introduces ML to develop predictive models to predict FTIR intensity, advancing beyond the purely experimental focus of these works.

Advanced ML techniques enable the prediction of FTIR sample intensities by estimating high-temperature conditions from low-temperature data. Such techniques include Linear Regression (LinR), partial least squares regression (PLSR), support vector regression (SVR),^8,9^ gradient-boosting regression (GBR), random forest (RF)^10,11^ and *k*-nearest neighbors (*k*-NN).^12^ By applying these techniques, it becomes possible to capture complex nonlinear relationships between experimental conditions and the resulting FTIR spectral data. These models effectively learn from the data patterns, thereby predicting new outcomes based on input conditions such as temperature and reaction time. Additionally, ML models provide rapid, real-time predictions, facilitating immediate decision-making and process control in various industrial and research applications. This automation reduces the need for manual spectral interpretation, saving time and resources while ensuring consistent, high-quality results. Moreover, ML-based soft sensors can adapt to new data over time, continuously improving their performance and scalability. These advantages make ML techniques indispensable for maximizing the potential of FTIR spectroscopy in chemical analysis and monitoring.^13^

Several studies have contributed significantly to advancing predictive modeling and FTIR spectroscopy for material analysis, providing a strong foundation for further exploration. Among these, Tefera et al.^14^ developed a Bayesian learning framework, a branch of ML, to model the reaction network of low-temperature visbreaking (150–400 °C) for partially upgrading oil sands bitumen. By analyzing FTIR spectroscopic data, Bayesian hierarchical clustering grouped chemically similar pseudospecies, while Bayesian network learning inferred causal relationships between these groups. This innovative framework captured reaction pathways effectively, demonstrating the potential for real-time process monitoring with minimal prior knowledge. However, its focus remained on understanding reaction networks rather than directly predicting the FTIR data itself or linking spectral data to property predictions. Expanding on this foundation, Tefera et al.^15^ also introduced a self-modeling multivariate curve resolution (SMCR) algorithm to monitor the thermal conversion of bitumen using FTIR. This approach emphasized automation and resolved spectral and concentration profiles, enabling insights into reaction mechanisms and underscoring its industrial applicability. Despite its practical effectiveness, the work primarily addressed decomposition and resolution rather than leveraging spectral data for predictive analysis. In parallel, Sivaramakrishnan et al.^2^ applied SMCR enhanced with particle swarm optimization (PSO) to analyze Athabasca bitumen thermal conversion. Their study focused on optimizing resolution quality and extracting detailed reaction pathways, particularly at varying temperatures. While this research advanced the resolution of pseudocomponents and clarified chemical transformations, it did not explore predictive modeling of spectral data. Beyond SMCR, Sivaramakrishnan et al.^16^ also demonstrated the use of ML to predict thermogravimetric data of asphaltenes extracted from deasphalted oil (DAO) doped with varying amounts of indene. Their study also utilized SVR, RF, and GBR models and observed that decision trees showed excellent prediction accuracy. This was an unexplored domain in DAO-TGA prediction, which we extent it to thermally cracked products of bitumen-FTIR prediction that offers new opportunities for predictive modeling and deeper insights into bitumen properties and reaction chemistry as well.

On the other hand, Ma et al.^17^ directed their efforts toward classifying bitumen types and aging states through FTIR spectroscopy and multivariate analysis. By identifying specific spectral regions linked to chemical changes over time, their work underscored the value of FTIR in chemical differentiation. Building on this, Weigel and Stephan^18^ extended the application of FTIR by correlating spectral data with physical properties such as viscosity and softening point, showcasing its predictive potential for functional characteristics. While both studies demonstrated the strengths of traditional chemometric techniques, their reliance on predefined spectral regions and linear assumptions constrained their ability to fully capture the nonlinear interactions inherent in bitumen’s complex behaviors. Together, these studies illustrate the evolution of analytical methods in bitumen research, progressing from understanding chemical transformations to linking spectral data with material properties and exploring predictive capabilities. However, while these works achieved remarkable advancements, each left specific gaps unaddressed. The emphasis on reaction pathways, automation, or limited predictive modeling, as seen in the works by Tefera and Sivaramakrishnan (mentioned in the previous paragraph), and the focus on traditional statistical methods, as demonstrated by Ma and Weigel (mentioned in this paragraph), highlight opportunities for further innovation.

Linear, tree-based and neural networks were applied to predict density functional theory (DFT) features (energy-related such as Gibbs energy, enthalpy, entropy, and electronic structure-related such as dipole moment, band gap) associated with 1031 entries of supramolecular structures consisting of dimer, trimer, and tetramer cyclics with and without heteroatoms in the work by Normatov et al.^19^ This clearly demonstrated that ML techniques were order of magnitudes quicker than the quantum chemistry-based DFT approach. Another interesting study conducted by Prof. Skorb’s group illustrated that gradient boosting algorithm showed the best performance in detecting antibiotics in milk.^20^ Artificial neural networks (ANN) were also used to assist in the design of electronic components such as diodes, capacitors, resistor, memristor allowing for the prediction of hydrogel compositions using 1 hidden layer and 12 nodes.^21^ It was very interesting to note that ML models such as SVR, *k*-NN, RF and GBR were also used to detect active and inactive inhibitors with 98% accuracy, for targeting cyclin-dependent kinase 2 (CDK2), which is primarily involved in tumorigenesis in the human body.^22^ Furthermore, a review of recent computational methods and machine learning applications to chemistry and material science fields such as accelerated design of flame-retardant polymeric nanocomposites, thermoelectric material property predictions, identification of copolymer microstructures based on microscopic images, discrimination of quartz genesis, anaerobic codigestion of glycerol and molasses, optimization of metallurgy-based parameters for enhancing mechanical properties of alumino-copper oxide composites, has been published by Novikov.^23^ These works explore the utility of ML-based approaches in various fields but not bitumen-based feedstock or its infrared spectra specifically.

Building on these advancements, our current research in this work addresses these critical gaps by integrating state-of-the-art ML models, notably GBR, RF and *k*-NN with Bayesian optimization for hyperparameter tuning. Unlike prior works, this study utilizes the full FTIR spectral data set to uncover complex nonlinear relationships, expanding the applicability of FTIR data analysis beyond traditional methods. Rather than relying on predefined spectral regions or focusing solely on reaction pathways, this work bridges chemical characterization with predictive property modeling, offering a dynamic and adaptable framework. The general approach to choose the hyperparameters in the ML models is visual trial and error by checking for a plateau in the prediction accuracies with varying the hyperparameters. One of the key novelties in this work is utilizing Bayesian optimization for choosing the hyperparameters, which has been shown before for landslide susceptibility mapping.^24^ By leveraging advanced ML techniques, the study not only enhances predictive accuracy but also establishes a streamlined methodology for real-time monitoring and optimization in industrial contexts. This integration of advanced computational tools with FTIR spectroscopy sets a new benchmark for precision in analyzing bitumen properties. It refines our understanding of how spectral features correlate with chemical and mechanical performance metrics, surpassing the limitations of prior studies. While previous works laid a strong foundation, this research propels the field forward, offering a comprehensive approach that unites chemical insights with predictive capabilities in a way that has not been achieved before. This signifies another key novelty of our work.

To advance this framework, the current investigation focuses specifically on employing ML techniques to predict FTIR intensities of products resulting from the noncatalytic thermal cracking of Athabasca bitumen, while simultaneously identifying the most accurate predictive models through Bayesian optimization. The data set employed consists of historical FTIR data collected under controlled reaction conditions spanning temperatures from 300 to 420 °C and reaction times ranging from 15 min to 27 h based on our previous experimental work.^1^ The aim is to not only achieve high predictive accuracy but also to explore the versatility of the models under varying conditions. This is accomplished through four distinct scenarios, each designed to rigorously test the models’ performance across diverse data sets:

1. Scenario 1 involves all 61,740 data points, employing an 80/20 train-test split with 10-fold Cross-Validation (CV) to evaluate overall model accuracy and generalizability.
2. Scenario 2 trains the models using data at temperatures of 25 °C, 350 °C, and 400 °C, and tests their predictive performance on unseen data at 300 °C, 380 °C, and 420 °C.
3. Scenario 3 reverses the training and testing groups by training the models on 350 °C, 380 °C, and 400 °C, while testing on 25 °C, 300 °C, and 420 °C.
4. Scenario 4 broadens the training set to include data at 25 °C, 300 °C, 350 °C, and 380 °C, and evaluates the models on temperatures of 400 and 420 °C which performs the training on low-temperature data to predict high-temperature data.

## 2 Methodology

### 2.1 Data Sets Used

This study applies ML techniques to predict FTIR intensities of products from the thermal cracking of bitumen. The experimental setup utilized Athabasca bitumen as the feedstock, processed in four batch microreactors. The reactors were heated using a fluidized sand bath, allowing precise temperature control. The reactions were conducted at varying temperatures of 25, 300, 350, 380, 400, and 420 °C. Reaction times varied for each temperature ranging from 15 min to 27 h. Postreaction, the products were categorized into gas, solid, and liquid phases. Solid particles were left to settle for 1 week, after which only the liquid fraction was used for FTIR analysis. Further details of the procedure are provided in Sivaramakrishnan et al.^1^ The number of data points for all temperatures are shown in Table 1, where the total number of data used for the analysis was 61,740 with the data points at 400 °C comprising 45.7% of the total data.

**Table 1:** Number of Data Points at Each Visbreaking Reaction Condition

temperature (°C)	data points
420	7056
400	28,224
350	10,584
380	10,584
420	7056
300	3528
Total	**61,740**

The ML techniques were applied on 4 different scenarios that emphasized different training and testing data points as shown in Table 2.

**Table 2:** Different Scenarios Used for Training and Testing Splitting of the Data Points from Visbreaking of Athabasca Bitumen for Prediction of FTIR Data

scenario #	train-test split
scenario 1	training and testing on all data points at all temperatures based on an 80/20 train-test split
scenario 2	training on temperatures of 25, 350, and 400 °C and testing on 300, 380, and 420 °C (random selection)
scenario 3	training on temperatures of 350, 380, and 400 °C and testing on 25, 300, and 420 °C (random selection)
scenario 4	training on temperatures of 25, 300, 350, and 380 °C (lower temperature training) and testing on 400 and 420 °C (higher temperature testing)

Ideally, scenario 4 is the main objective of this work, where the ML techniques are utilized to develop a soft-sensor that is trained on data obtained at lower temperatures and subsequently used to predict the FTIR intensities at higher temperatures. By achieving this, the behavior of the thermally cracked products of bitumen can be effectively modeled without the need for extensive high-temperature characterization using offline instrumentation.

### 2.2 Software Tools Used

#### 2.2.1 Data Preprocessing and Regression

This study leverages a comprehensive suite of libraries in Python to perform data manipulation, model training, evaluation, and optimization. The data was preprocessed to better fit the ML algorithm. To reshape the DataFrame from a wide format to a long format, the function “*data.melt*” was used from the pandas library for data manipulation and analysis. This is particularly useful for analysis to improve data organization and prepare it for ML implementation. “*Numpy*” library is utilized for numerical operations, offering powerful capabilities for array manipulations and mathematical computations. For splitting the data set into training and testing subsets, the “*train_test_split*” function from “*sklearn.model_selection*” is employed to split the data into training and testing sets as illustrated in Table 2 for each scenario. This function ensures that the data is divided in a manner that allows for robust training and evaluation of the models. The study explores several regression techniques, including “*LinearRegression*” from “*sklearn.linear_model*”, which serves as a baseline model due to its simplicity and interpretability. SVR from “*sklearn.svm*” is used to incorporate SVR, known for its effectiveness in capturing nonlinear relationships. The “*KNeighborsRegressor*” from “*sklearn.neighbors*” is applied for instance-based learning, which predicts the target by considering the nearest neighbors. Ensemble methods such as “*RandomForestRegressor*” and “*GradientBoostingRegressor*” from “*sklearn.ensemble*” are also utilized. These methods are renowned for their ability to improve predictive performance by combining the strengths of multiple base learners. Additionally, “*PLSRegression*” from “*sklearn.cross_decomposition*” is used to handle data sets with multicollinearity and to perform dimensionality reduction. To visualize the results effectively, the “*altair*” and “*matplotlib*” libraries are employed, providing a declarative framework for creating interactive and interpretable visualizations. Moreover, for three-dimensional visualization, particularly to depict complex data structures and relationships, “*Axes3D*” from “*mpl_toolkits.mplot3d*” is utilized. This tool allows for the creation of 3D plots, enhancing the ability to interpret multidimensional data. The “*time*” library is used to measure the execution time of different models, offering insights into their computational efficiency and speed.

#### 2.2.2 Cross-Validation and Root-Mean-Square Error (RMSE)

To enhance the predictive accuracy and robustness of the ML models, a 10-fold cross-validation (CV) technique is employed using the “*cross_val_score*” function and “*KFold*” from “*sklearn.model_selection*”. This method divides the data set into ten subsets, with each subset used as a validation set while the remaining nine subsets are used for training. The use of 10-fold CV is particularly advantageous because it offers a balanced trade-off between computational efficiency and statistical reliability. Research consistently demonstrates that 10-fold CV minimizes bias and variance, making it a preferred method for model evaluation.^25^ By averaging the results across all folds, this technique reduces the risk of overfitting, providing a more accurate and generalizable estimate of model performance compared to simpler validation techniques.^26^ When the CV Root Mean Squared Error (RMSE) and test RMSE values are similar, it indicates that the model generalizes well and is likely a good fit. Consequently, 10-fold CV is favored for its ability to provide a comprehensive evaluation while maintaining practical feasibility.^27^

#### 2.2.3 Graphical Hyperparameter Tuning and Bayesian Optimization

In the hyperparameter tuning process, a combination of libraries and techniques was utilized to efficiently explore different model configurations in the form of graphical visualizations. LinR, PLSR and SVR were not explored using this process as they proved to be insufficient and time-consuming as will be shown in the results section. Thus, each algorithm was run separately for each of the 3 ML Techniques (RF, GBR and *k*-NN) for each scenario. The data set was then divided into training and testing sets using the “*test_train_compound*” function corresponding to the appropriate splitting conditions that characterizes each scenario as previously shown in Table 2. To evaluate different hyperparameter combinations, lists of possible values of the hyperparameters for each technique were created accordingly. Nested loops were used to iterate over each combination of the appropriate hyperparameter. For each combination, an ML model was trained with those parameters, and the corresponding training and testing accuracies were recorded. This manual approach systematically explored the performance of the model across a wide range of hyperparameter values. After the training loop, a DataFrame was created to store the results, and the ‘melt’ function was used to reformat it for easier visualization. The *Altair* library was employed to generate graphs. A base chart was created, and faceting was used to produce separate charts for each value of one hyperparameter, illustrating how accuracy varies with another hyperparameter. This method facilitated the comparison of the effects of different hyperparameter values on model performance.

This study also integrates the “*BayesianOptimization*” function from the “*bayes_opt*” library for hyperparameter tuning to systematically explore the hyperparameter space. This method builds a probabilistic model of the objective function to select the most promising hyperparameters for evaluation, making it more efficient than traditional grid or random search methods. In addition, to ensure reproducibility, a random seed is used with the “*random_state*” parameter by controlling the randomness in data splitting, shuffling, and model initialization processes, so that the results remain consistent across different runs. Furthermore, *R* ^2^ (coefficient of determination), RMSE and overfitting tendency were used as performance metrics to evaluate the accuracy of the ML models from “*sklearn.metrics*” library including “*mean_squared_error*”, “*make_scorer*”, and “*r2_score’*. This study employs a diverse array of libraries to ensure robust data manipulation, model training, visualization, evaluation, and optimization, ultimately leading to more accurate and reliable predictive modeling outcomes by comparing several ML techniques under different scenarios that highlight diverse splitting of the training and testing data in order to analyze how well the models generalize to unseen data. Additionally, the integration of advanced visualization techniques aids in the clear and effective presentation of results.

### 2.3 Brief Theory of ML Techniques and Effects of Hyperparameters

The readers are referred to previous works for theory on linear regression using ordinary least-squares,^28,29^ and PLSR.^30,31^ Furthermore, principles of *k*-nearest neighbors (*k*-NN) and SVR are elaborated in the Supporting Information document.

#### 2.3.1 Gradient Boosted Regression (GBR)-Based Decision Trees

GBR is an ensemble learning method used for regression tasks. It builds strong models by iteratively combining weak learners, i.e., decision trees. GBR minimizes a loss function using gradient descent, where each new tree aims to correct the errors of the previous model.^32,33^ The squared error loss is commonly used for regression tasks and is defined as^34^

1 $$L\left(y,ŷ\right)=\sum_{i=1}^{N}{\left(y_{i}-{ŷ}_{i}\right)}^{2}$$

where *y*_*i*_ represents the true target value, *ŷ*_*i*_ is the predicted value, and ***N*** is the number of observations. At each iteration, GBR updates predictions by training a decision tree to approximate the negative gradient of this loss function.^35^ The gradient at the next iteration is calculated as

2 $$-\frac{\partial L\left(y,ŷ\right)}{\partial {ŷ}_{i}}=y_{i}-{ŷ}_{i}=r_{i}$$

For squared error loss, the gradient simplifies to the residuals *r*_*i*_, making the residuals the target for the weak learner *h*_*m*_ (*x*). The weak learner is trained to minimize the squared error between predicted and actual residuals^36^

3 $$h_{m}\left(x\right)=\arg\;\min\sum_{i=1}^{N}{\left(r_{i}-h_{m}\left(x_{i}\right)\right)}^{2}$$

The current prediction, *ŷ*_*m*_ is updated by adding the contribution of the weak learner, *h*_*m*_ (*x*) scaled by η, the learning rate (LR) to the previous iteration’s prediction, *ŷ*_*m*–1_ ^37^

4 $${ŷ}_{m}={ŷ}_{m-1}+\eta h_{m}\left(x\right)$$

This process is repeated until a specified stopping criterion has been reached. One such criterion is a specified number of weak learners, controlled by the hyperparameter *n_estimators* (***n***). Other hyperparameters in GBR include the LR and maximum depth of trees (*D*).^38^ A smaller LR requires more trees for convergence, while a larger rate speeds up learning but may lead to overfitting. A deeper tree (higher *D*) captures more complex patterns but may increase the risk of overfitting, while a shallower tree (lower *D*) helps prevent overfitting by acting as a regularizer.^39^ The optimal configuration balances model accuracy and computational efficiency, and Bayesian optimization is often used to identify the best combination of these hyperparameters.^40^

#### 2.3.2 Random Forest Regression (RF)-Based Decision Trees

RF is a powerful ensemble learning algorithm commonly used for regression and classification tasks. It builds multiple decision trees during training and combines their predictions to form a single, robust model.^41^ RF introduces randomness into the training process through bootstrapping and feature selection, which increases the diversity among the individual trees and reduces overfitting.^42^ Unlike single decision trees, RF achieves better stability and generalization by relying on the collective predictions of many weak learners.^43^ For regression tasks, RF predicts by averaging the outputs of all decision trees. The final prediction for an input ***x*** is given by^44^

5 $$ŷ=\frac{1}{T}\sum_{t=1}^{T}{ŷ}_{t}$$

where *T* is the total number of trees, *ŷ*_*t*_ is the prediction from the *t*th tree. The algorithm uses variance reduction as the criterion for evaluating splits, aiming to increase homogeneity within each node. The variance σ^2^ of the target values in a node is calculated as^45^

6 $$\sigma^{2}\left(D\right)=\frac{1}{N}\sum_{i=1}^{N}{\left(y_{i}-\mathrm{y̅}\right)}^{2}$$

where *y*_*i*_ are the target values for the data points in the node, *y̅* is the mean of the target values, and *N* is the number of samples in the node. The goal of each split is to minimize the weighted average of the variances of the child nodes, which is referred to as variance reduction^46^

7 $$\Delta \sigma^{2}=\Delta \sigma_{parent}^{2}-\left(\frac{N_{L}}{N}\sigma_{L}^{2}+\frac{N_{R}}{N}\sigma_{R}^{2}\right)$$

where Δσ_parent_^2^ is the variance of the parent node, and σ_L_^2^ and σ_R_^2^ are the variances of the left and right child nodes, with *N*_*L*_ and *N*_R_ being the respective number of samples in those nodes.^47^

RF’s performance is influenced by hyperparameters such as the number of trees (*n*) and the maximum depth (*D*) of each tree. A larger number of trees typically improves performance by reducing variance, while deeper trees (more *D*) allow for capturing more detailed patterns but may lead to overfitting. On the other hand, shallower trees are less prone to overfitting but may underfit more complex relationships. As mentioned in the introduction, the optimal configuration of these parameters is usually determined through determined through visual approach by analyzing the prediction accuracies for different *n* and *D* values but this work utilized Bayesian optimization to choose these hyperparameters in order to improve model accuracy and balance it with computational efficiency.^24^

### 2.4 Evaluation Metrics

#### 2.4.1 Coefficient of Determination (*R* ^2^)

*R* ^2^ is one of the most widely used evaluation metrics in regression models. It is a powerful parameter that quantifies the proportion of variance in the dependent variable that is explained by the independent variables in the model. The formula for *R* ^2^ is given by^48^

8 $$R^{2}=\frac{\sum_{i=1}^{n}{\left(y_{i}-{ŷ}_{i}\right)}^{2}}{\sum_{i=1}^{n}{\left(y_{i}-\mathrm{y̅}\right)}^{2}}$$

where *y*_*i*_ is the actual (experimental) value, *ŷ*_*i*_ is the predicted value, and *y̅* is the mean of the actual values. This metric ranges from 0 to 1, where a value of 1 indicates that the model explains all the variance, and a value of 0 means that the model explains none of the variance. The *R* ^2^ is particularly useful in evaluating how well a model fits the data and whether the independent variables have a meaningful relationship with the dependent variable. It is a simple yet powerful metric that helps in determining the overall effectiveness of a regression model in capturing the patterns within the data. In this study, the *R* ^2^ for the training and testing sets will be referred as training accuracy and testing accuracy, respectively, as it directly quantifies how well the model accounts for the variability of the target variable. The higher the accuracy value, the better the model is at explaining the observed data. It offers an intuitive and interpretable metric for assessing model performance.

#### 2.4.2 RMSE

RMSE is another critical metric used to evaluate the performance of regression models. RMSE measures the average magnitude of the errors between predicted and actual values by taking the square root of the average squared differences. The formula for RMSE is^49^

9 $$RMSE=\sqrt{\frac{1}{n}\sum_{i=1}^{n}{\left(y_{i}-{ŷ}_{i}\right)}^{2}}$$

where ***y***_***i***_ and *ŷ*_*i*_ are the actual and predicted values, respectively, and *n* is the number of data points. RMSE provides an estimate of the model’s prediction accuracy by penalizing larger errors more heavily due to the squaring of the residuals. This makes it particularly useful when large errors are undesirable or when the scale of the data matters. RMSE is also expressed in the same units as the original experimental/predicted data, making it interpretable in the context of the problem. A lower RMSE indicates better model performance, as it implies that the model’s predictions are closer to the actual values.

#### 2.4.3 Overfitting Tendency

The overfitting tendency can be effectively evaluated by comparing the *R* ^2^ values of the training and testing data sets. This approach highlights how well the model generalizes to unseen data and can be expressed as the difference between *R* ^2^ of the training set and *R* ^2^ of the testing set. When a model exhibits a high *R* ^2^ on the training data (*R*_Train_^2^) but a significantly lower *R* ^2^ on the testing data (*R*_*test*_^2^), it suggests that the model is overfitting. Overfitting occurs when a model learns to memorize the noise or specific patterns in the training data, resulting in poor generalization to new, unseen data.^50^ The formula for evaluating overfitting tendency can be given as

10 $$overfitting\;tendency=R_{Train}^{2}-R_{Test}^{2}$$

This metric provides valuable insight into how well a model is likely to perform on future data. A large gap between the training and testing *R* ^2^ values indicates that the model has likely become too complex and is not generalizing well, thus demonstrating overfitting. This metric is especially useful in guiding the adjustment of model complexity and the selection of regularization techniques to avoid overfitting. By minimizing the difference between training and testing *R* ^2^, one can achieve better generalization, ensuring that the model performs consistently on both seen and unseen data.

### 2.5 Bayesian Optimization

Bayesian optimization is a robust technique for optimizing ML models by efficiently selecting hyperparameter combinations to maximize performance. It is particularly useful when hyperparameter evaluations are computationally expensive.^51^ In Bayesian optimization, the objective function quantifies model performance based on selected hyperparameters, often using a metric like RMSE. Since the “*bayes_opt*” library in Python maximizes functions by default, the objective function is typically defined as the negative RMSE, allowing for optimization through maximization. The optimization process relies on an acquisition function, which guides the search for the next set of hyperparameters to evaluate. The acquisition function balances two key strategies: exploration and exploitation.^52^ Exploration focuses on regions with high uncertainty, while exploitation targets regions that have already demonstrated good performance. One common acquisition function used in this process is the upper confidence bound (UCB), defined as

11 a(x)=μ(x)+κσ(x)

where *a*(*x*) is the acquisition function, μ (*x*) is the predicted mean of the objective function for a given set of hyperparameters *x*, σ­(*x*) is the predicted standard deviation (uncertainty) of the objective function at *x* and *k* is a tunable parameter that controls the trade-off between exploration and exploitation. Larger values of κ encourage exploration, while smaller values prioritize exploitation. The default value of κ = 2.576 corresponds to a 99% confidence interval in a standard normal distribution, helping balance the trade-off. The acquisition function operates over a surrogate model, which is a probabilistic approximation of the objective function based on prior evaluations. This surrogate model predicts both the mean and uncertainty of the objective function for unexplored hyperparameter combinations. By maximizing the acquisition function^38^

12 $$x_{n+1}=\arg\;\max a\left(x\right)$$

The next set of hyperparameters ***x***_***n*+1**_ is selected, aiming to either explore areas of high uncertainty or exploit areas with promising objective function predictions. Bayesian optimization operates iteratively, with each iteration involving a sequence of steps that systematically refine the search for optimal hyperparameters.^53^ First, the acquisition function selects the next set of hyperparameters to evaluate. Once the hyperparameters are proposed, the objective function, represented by the negative RMSE is computed by training the ML model and evaluating its performance on a test set. The results of this evaluation are then used to update the surrogate model, which approximates the objective function. This refinement improves the model’s ability to predict the objective function across the hyperparameter space. The process is repeated iteratively, with the stopping criterion in this case being a predefined number of iterations.^54^

Thus, Bayesian optimization is highly effective for hyperparameter tuning because it automates the search for optimal values, eliminating the need for manual trial-and-error. By efficiently exploring the hyperparameter space and focusing evaluations on promising regions, it accelerates the optimization process. Furthermore, Bayesian optimization adapts dynamically based on new information, refining the search progressively, which makes it well-suited for high-dimensional and complex parameter spaces.^52^

## 3 Results and Discussion

### 3.1 Results from Linear Regression (LinR), Partial Least-Squares (PLSR) and SVR

The results presented in Table 3 correspond to those obtained for all scenarios by employing LinR, PLSR, and SVR, which were explored by tuning various hyperparameters to assess their performance across different scenarios. The hyperparameters shown are the best-tuned ones giving the highest prediction accuracy for that scenario. LinR does not have any hyperparameters to tune since it is the most basic form of regression using ordinary least-squares, while PLSR was tested with 2 and 3 latent components (also called factors) to examine the effect of dimensionality reduction. SVR models were evaluated with both the linear kernel and the RBF kernel, where the regularization parameter (*C*), the scale/kernel parameter (γ), and the tolerance parameter (ε) were explored. These hyperparameter configurations aimed to optimize the models for the given data. However, these models were ultimately eliminated from further consideration due to their poor performance and computational inefficiencies. Note that accuracy means *R* ^2^.

**Table 3:** All Scenarios: Performance of LinR, PLSR and SVR

model	hyperparameters			test accuracy	train accuracy	overfit trend	train RMSE	test RMSE	time (s)
Scenario 1									
LinR	no hyperparameters			0.0917	0.0969	0.0052	3.4672	3.4566	0.6
PLSR	2 components			0.0917	0.0969	0.0052	3.4672	3.4566	0.7
PLSR	3 components			0.0917	0.0969	0.0052	3.4672	3.4566	0.37
SVR (Linear kernel)	*C* = 1	ε = 0.01		0.0917	0.0969	0.0052	3.4672	3.4566	29,363
SVR (RBF)	*C* = 10	ε = 0.01	γ = 0.1	0.9399	0.9979	0.058	0.1654	0.8894	27,487
Scenario 2									
LinR	no hyperparameters			0.016	0.13	0.11	3.52	3.37	0.46
PLSR	2 components			0.016	0.13	0.11	3.52	3.37	0.36
PLSR	3 components			0.016	0.13	0.11	3.52	3.37	0.37
Scenario 3									
LinR	no hyperparameters			0.094	0.05	–0.04	3.50	3.52	0.37
PLSR	2 components			0.094	0.05	–0.04	3.50	3.52	0.38
PLSR	3 components			0.094	0.05	–0.04	3.50	3.52	0.43
Scenario 4									
LinR	no hyperparameters			0.045	0.12	0.07	3.58	3.41	0.26
PLSR	2 components			0.045	0.12	0.07	3.58	3.41	0.35
PLSR	3 components			0.045	0.12	0.07	3.58	3.41	0.34

LinR which is a basic model that assumes a linear relationship between input variables and the target variable, showed very low test accuracy across all scenarios, with values consistently around 9%. This suggests that LinR is not suitable for the complexity of the data set, as it fails to capture nonlinear relationships and interactions effectively. Similarly, PLSR, which uses principal components to reduce the dimensionality of the data before fitting the model, also exhibited the same performance across both 2-component and 3-component configurations, with test accuracies consistently around 9%. Since there are only 3 features in this data set, performing PLSR with 3 components essentially behaves like linear regression, as the dimensionality reduction step offers no real benefit. Thus, the behavior of LinR and PLSR across different configurations was largely the same, suggesting that neither model can effectively handle the data set’s complexity.

For SVR, both the linear kernel and the RBF kernel demonstrated significant computational inefficiencies. The time required for each run was around 7 to 8 h per single reading which is too computationally expensive and impractical, particularly when compared to other models that provide faster processing times and much better performance. Given the infeasibility of these techniques in terms of both accuracy and computational cost, they will no longer be considered in further analysis.

### 3.2 Scenario-Wise Graphical Hyperparameter Tuning for RF, GBR and *k*-NN Models

#### 3.2.1 Scenario 1: RF

Figure 1 provides a detailed analysis of the RF models’ performance in Scenario 1, utilizing an 80/20 train-test split on all data points with 10-fold CV. The graphs display the accuracy of RF models with different numbers of estimators (50, 100, 200, and 300) and varying maximum depths (10–25). For all combinations of hyperparameters, the accuracy is quite high (above 97%) which indicates that RF is a suitable ML technique for this data set. Training accuracy exceeds testing accuracy at depths higher than 15, indicating some degree of overfitting. As the number of estimators increases, the trend of both training and testing accuracy are similar indicating that number of estimators does not have a significant impact on the accuracy. Lower depths (10–15) undergo rapid accuracy increases, but beyond a depth of 20, the rate of improvement diminishes, suggesting no further benefit from increasing model complexity. The testing accuracies at a depth of 20 for all facets are shown in the figure and these indicate that the accuracies are quite similar to each other with only a difference of 0.04% and 0.05% between the highest accuracy and the others. Consequently, the best RF model for scenario 1 is the configuration with 100 estimators and a depth of 20 which achieve the high testing accuracy of 99.36% with minimal overfitting.

**Figure 1:**
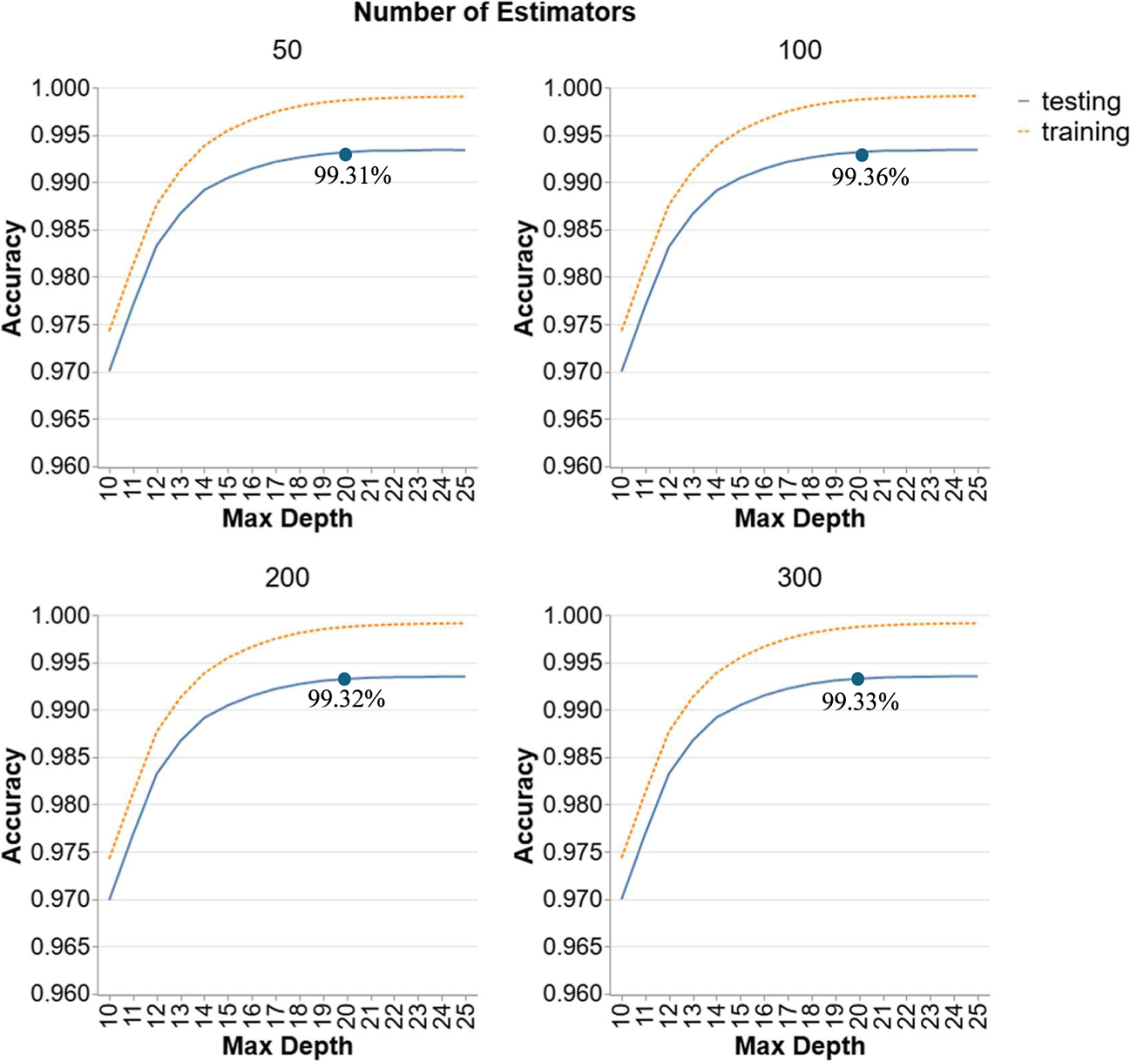
Hyperparameter tuning for RF models in Scenario 1.

#### 3.2.2 Scenario 1: GBR

Figure 2 illustrates the accuracy of GBR models for scenario 1 with different numbers of estimators (100, 150, 200, and 300) and varying depths (7 to 24) for two LRs (0.2 and 1). Generally, all hyperparameter configurations achieve accuracies above 98.8%, reflecting improved performance over RF for this data set. In addition, the same trend is observed in all facets suggesting that the two hyperparameters, LR and D, have the most significant effect on the accuracy. However, similar to RF, N has only a small effect in enhancing the accuracy. It can be observed that at lower depths, testing accuracy remains relatively high and stable, particularly for learning rate of 0.2. As depth increases beyond 10, testing accuracy slightly declines especially for learning rate of 1, which drops to around 98.8% at deeper levels, indicating less accuracy and some overfitting. Increasing the number of estimators from 100 to 300 slightly enhances testing accuracy from 99.59% to 99.66% at the optimal depth of 9 but does not prevent the decline in performance at higher depths where the highest accuracy of each number of estimator is indicated on the graph. Thus, the best GBR model for scenario 1 is the configuration with 300 estimators and a learning rate of 0.2 with the optimal testing accuracy of 99.66% observed at a depth of 9.

**Figure 2:**
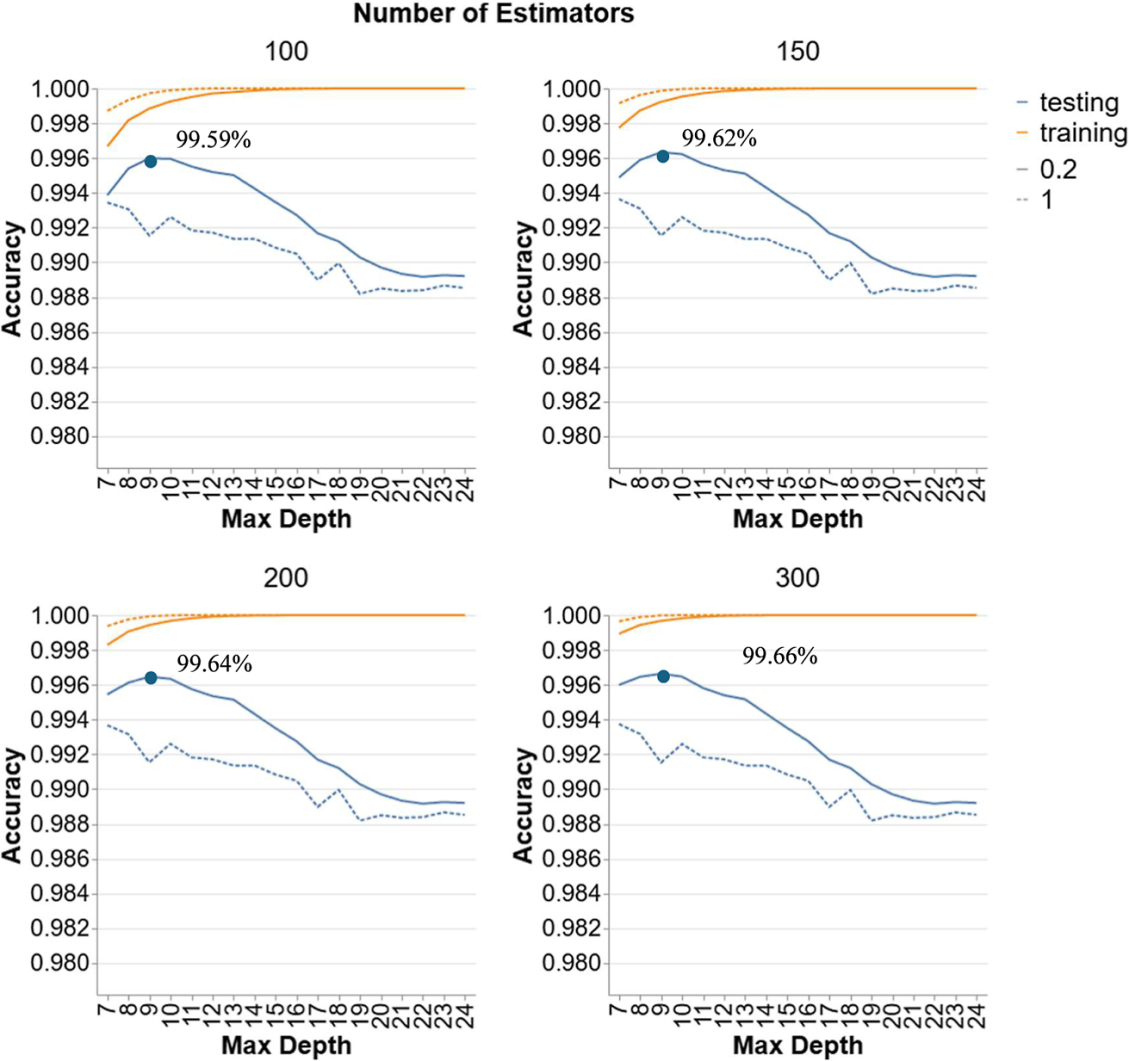
Hyperparameter tuning for GBR models in Scenario 1.

#### 3.2.3 Scenario 1: *k*-NN

Figure 3 depicts the performance of *k*-NN models in Scenario 1, with different leaf sizes (5, 10, 20, 30) and varying numbers of neighbors (1 to 10). Across all leaf sizes, the accuracy values indicated on the graph are identical indicating that leaf size has no impact on the testing accuracy. However, a peak is observed at number of neighbors of 2 for all leaf sizes suggesting that this hyperparameter has significant impact on the testing accuracy. Beyond this point, testing accuracy declines steadily as the number of neighbors increases, suggesting reduced model performance. This pattern indicates that a smaller number of neighbors helps capture local patterns more effectively, enhancing generalization and testing accuracy. As the number of neighbors grows, the model likely averages over more distant points, leading to decreased sensitivity to local data variations and, consequently, lower accuracy. As such, the best *k*-NN model for scenario 1 is the configuration with 2 neighbors and a leaf size of 5 since leaf size does not affect the accuracy, thus selecting a smaller leaf size is more appropriate to decrease model complexity. This model achieves a testing accuracy of 99.58%.

**Figure 3:**
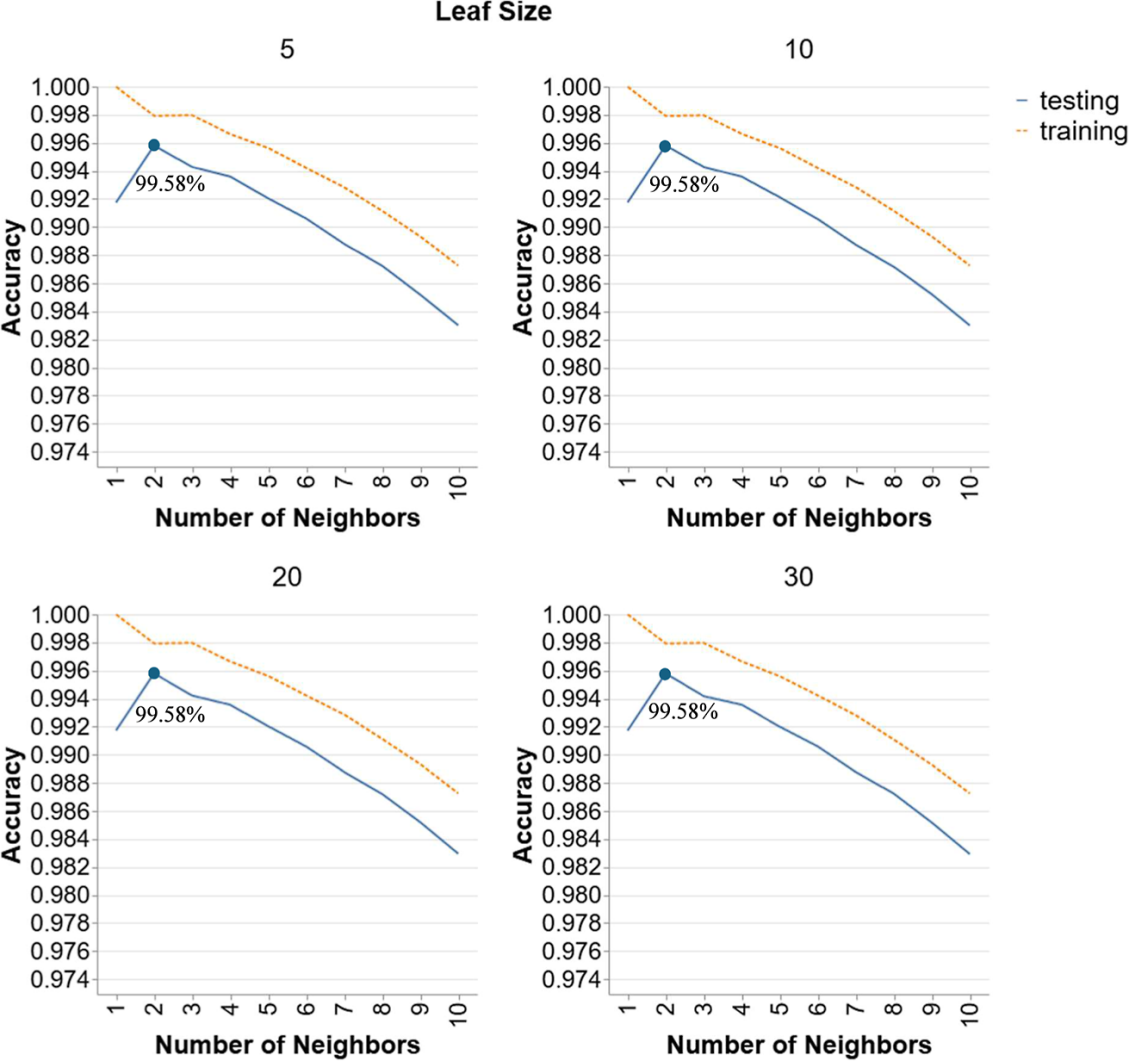
Hyperparameter tuning for k-NN models in Scenario 1.

#### 3.2.4 Scenario 2: RF

Figure 4 portrays the performance of RF models in Scenario 2 with different numbers of estimators (100, 200, 300, and 400) and varying maximum depths (5 to 15). The average accuracies in this scenario are still quite high but there is a slight decline compared to scenario 1 due to the data set chosen for the training and testing. Scenario 1 involved an 80/20 ratio of train-test split on all the data points. Whereas scenario 2 performed training on specific temperatures of 25, 350, and 400 °C and testing on the rest of the temperatures (300, 380, and 420 °C). The training set comprises 65% of the data, none of which are present in the testing set. The goal of this scenario is to assess how well the ML models can predict data for temperatures that were not included in the training set. As with the previous scenario, the behavior of the accuracy curve in the RF model remains unaffected by number of estimators. The testing accuracy peaks at a depth of 8 and then plateaus, while the accuracies at this peak depth are almost the same for all N ranging between 94.33% to 94.37%. This trend suggests that while deeper trees can capture more complexity in the training data, they do not necessarily translate to better performance on unseen data. The stable testing accuracy beyond certain depths indicates that the model’s ability to generalize does not benefit from additional complexity. Therefore, an optimal configuration for RF in Scenario 2 would involve a depth of 8 and a sufficient number of estimators of 300 to achieve the best testing accuracy of 94.37%.

**Figure 4:**
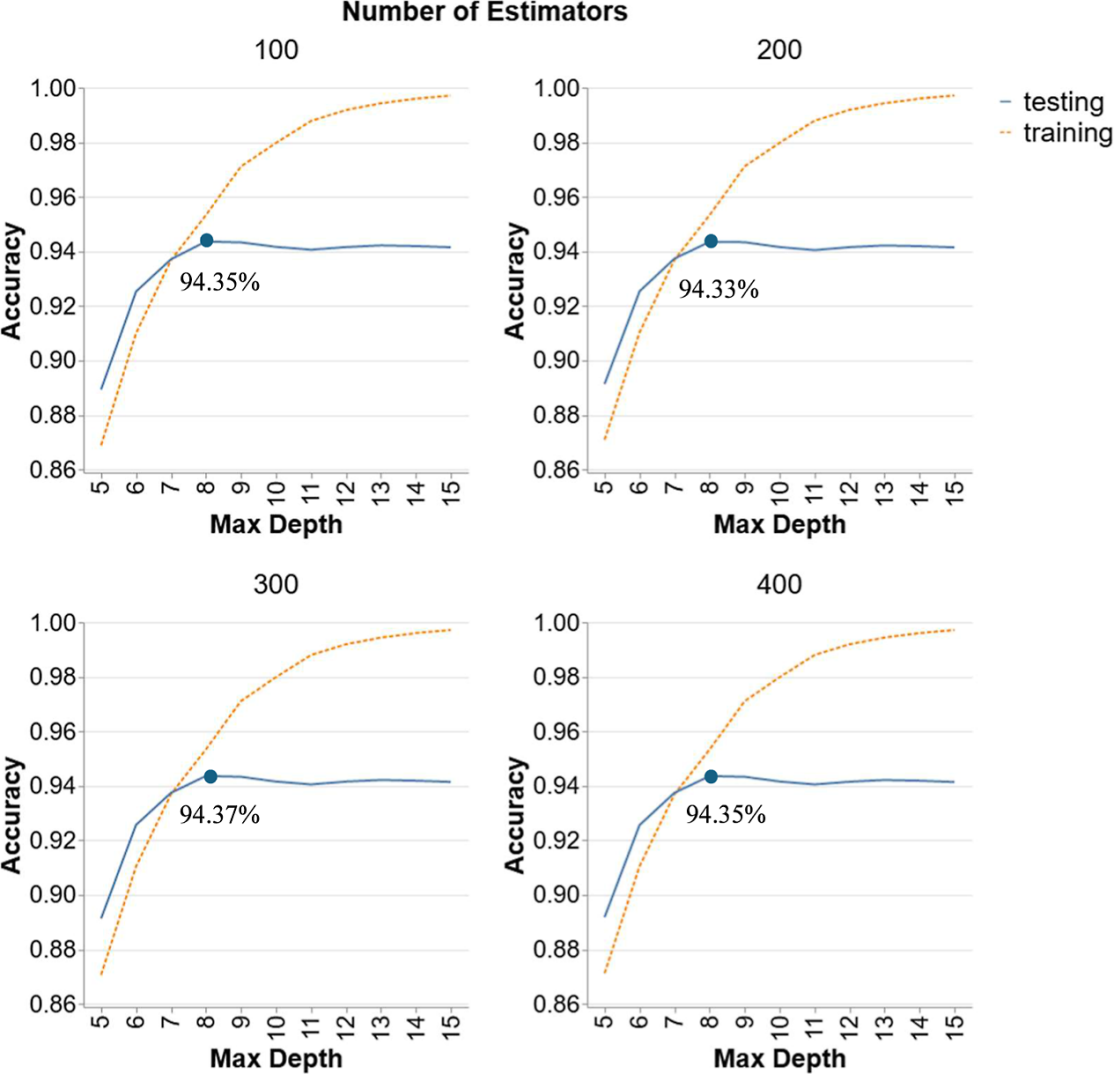
Hyperparameter tuning for RF models in Scenario 2.

#### 3.2.5 Scenario 2: GBR

Figure 5 highlights the performance of GBR models in Scenario 2 with different numbers of estimators (30, 50, 60, 100) and varying maximum depths (7–17) for different learning rates (0.1 and 1). Increasing the number of estimators from 30 to 100 does not significantly impact testing accuracy, which stabilizes around the same values, reinforcing the idea that beyond a certain point, additional estimators provide decreasing benefits in terms of accuracy improvement. Similar to RF, the accuracy peaks at a depth of 8 with a similar range of accuracies between 94.04% and 94.22%. For models with a higher LR of 1, testing accuracy is generally lower and exhibits more fluctuation, suggesting that the higher LR may cause the model to overfit quickly to the training data, reducing its generalization capability. Specifically, the highest testing accuracy is observed with a depth of 8, particularly for models with a learning rate of 0.1 and 50 estimators with a testing accuracy of 94.22%, indicating that these settings capture the complexity of the data effectively without overfitting.

**Figure 5:**
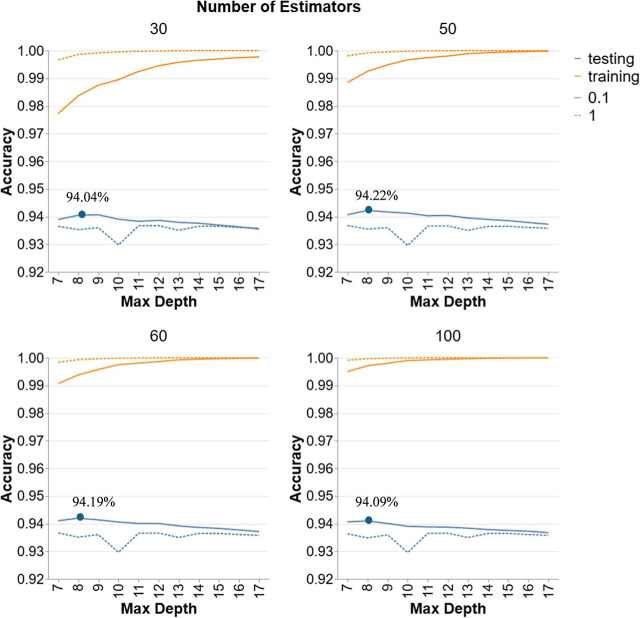
Hyperparameter tuning for GBR models in Scenario 2.

#### 3.2.6 Scenario 2: *k*-NN

Figure 6 showcases the performance of *k*-NN models in Scenario 2 for leaf size 5, 20, 30, and 50 and number of neighbors from 1 to 10. Similar to *k*-NN’s behavior in scenario 1, the figure shows consistent accuracy values across all leaf sizes, implying that leaf size does not influence testing accuracy. In contrast, a noticeable peak occurs at a number of neighbors equal to 7 for every leaf size, highlighting the significant effect of this hyperparameter on testing performance. This indicates that *k*-NN’s generalization ability in this scenario is not significantly affected by variations in leaf size. The peak performance at 7 neighbors suggests an optimal balance where the model effectively captures local data patterns without averaging over too many distant points, which would dilute the influence of nearer, more relevant neighbors. Thus, the best performing hyperparameter configuration for this model is with 7 neighbors and a leaf size of 5 achieving an accuracy of 91.96%.

**Figure 6:**
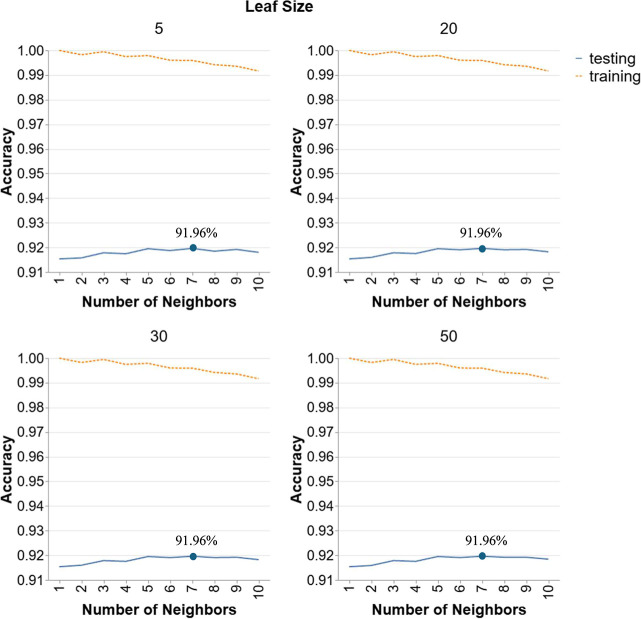
Hyperparameter tuning for *k*-NN models in Scenario 2.

#### 3.2.7 Scenario 3: RF

Following a parallel approach to scenario 2, scenario 3 is trained on temperatures of 350, 380, and 400 °C, and tested on temperatures of 25, 300, and 420 °C. Coincidentally, this setup accounts for 80% of the data, similar to Scenario 1, though the approach differs. While Scenario 1 involved an 80/20 random train-test split across all data points, Scenario 3 specifically tests the model on data points that were excluded from the training set, following the same general approach as Scenario 2. The objective here is to evaluate the performance of the ML models on unseen temperature data, ensuring a robust test of their predictive capabilities. Figure 7 presents the performance of RF models in Scenario 3 with numbers of estimators of 100, 150, 250, and 300 and varying maximum depths (7–20). Testing accuracy starts at around 73% accuracy and increases gradually with depth, but the improvement plateaus around a maximum depth of 15. Beyond this point, further increases in depth do not significantly enhance testing accuracy, which stabilizes around 75.2%. The number of estimators also influences the accuracy, with 150 estimators showing slightly better performance than others. However, the overall impact is modest, indicating that increasing the number of trees beyond a certain point yield declining results in terms of testing accuracy improvement. The highest testing accuracy is observed at a maximum depth of 15 with 150 estimators, stabilizing at approximately 75.27%. This suggests that while deeper trees and more estimators improve the model’s performance, the benefit levels off, highlighting the importance of adjusting model complexity to avoid overfitting and ensure good generalization to unseen data.

**Figure 7:**
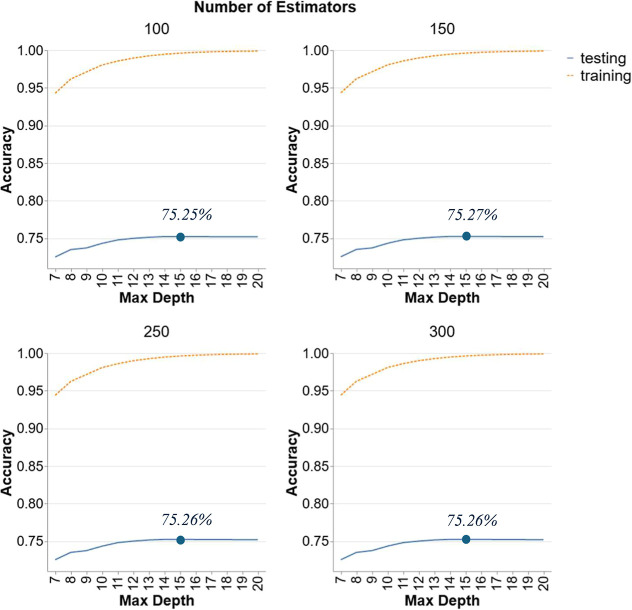
Hyperparameter tuning for RF models in Scenario 3.

#### 3.2.8 Scenario 3: GBR

Figure 8 features the performance of GBR models in Scenario 3. Each graph shows results for different numbers of estimators (100, 200, 300, 400) and two learning rates (0.5 and 1). GBR performed superiorly well compared to RF’s performance with accuracies ranging from 91.33% and reaching 91.9%. For both learning rates, testing accuracy starts low at a depth of 1, reaching a peak at a depth of 2, then declining sharply as depth increases, reaching a minimum around depths of 6, before improving and stabilizing. The highest testing accuracy for a learning rate of 0.5 is around 91.9%, observed at lower depths, indicating that simpler models generalize better to the testing data in this scenario. In contrast, models with a learning rate of 1 follow the same trend of 0.5 but with slightly lower accuracies. This suggests that a higher learning rate may cause the model to overfit the training data quickly and struggle to generalize to the testing data. The number of estimators (ranging from 300 to 400) does not significantly alter the overall pattern observed. While more estimators help maintain high training accuracy, their impact on testing accuracy is minimal, reinforcing the importance of optimal depth and learning rate oversimply increasing the number of estimators. Consequently, the optimal hyperparameter configuration for this model is with 200 estimators, a depth of 2 and an LR of 0.5 achieving an accuracy of 91.9%.

**Figure 8:**
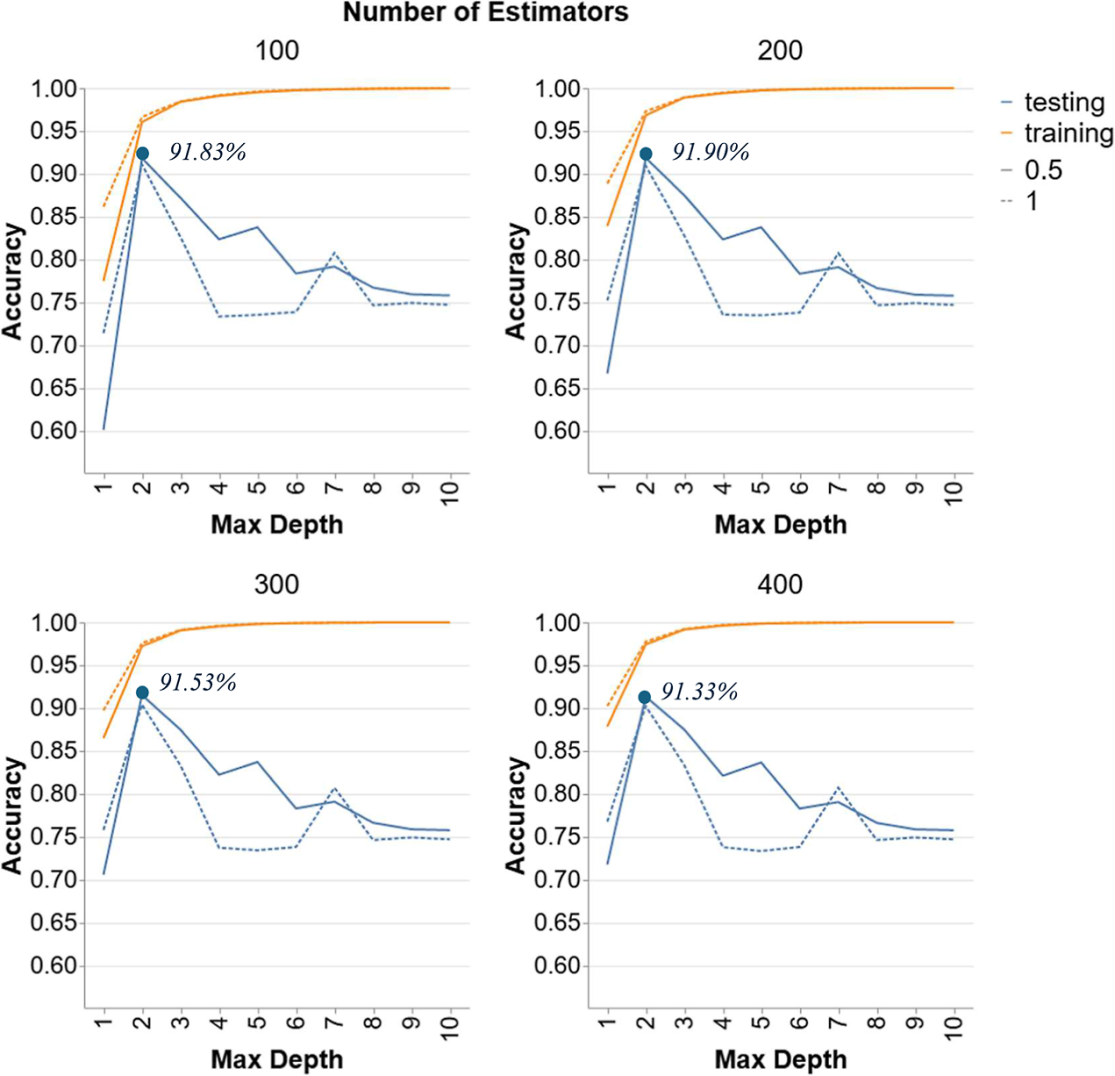
Hyperparameter tuning for GBR models in Scenario 3.

#### 3.2.9 Scenario 3: *k*-NN

Figure 9 shows the performance of *k*-NN models in Scenario 3 for leaf size of 5, 10, 30, and 50 and number of neighbors from 1 to 10. The testing accuracy remains relatively stable and low across different numbers of neighbors and leaf sizes, hovering around 74%. There is minimal variation in testing accuracy regardless of the number of neighbors, indicating that increasing the number of neighbors does not significantly enhance the model’s ability to generalize to unseen data in this scenario. The leaf size also appears to have little impact on testing accuracy, as the performance curves are nearly identical across all examined sizes. The overall low testing accuracy suggests that *k*-NN may not be well-suited for this scenario, where the training and testing temperature ranges are quite different. This lack of sensitivity to the number of neighbors and leaf size highlights the inherent limitation of *k*-NN in handling the variability and complexity of this particular data set. Thus, the best performing model will be chosen based on the smallest parameters leading to less complex models; that is, *N* = 2 and LS = 5 achieving a testing accuracy of 73.6%.

**Figure 9:**
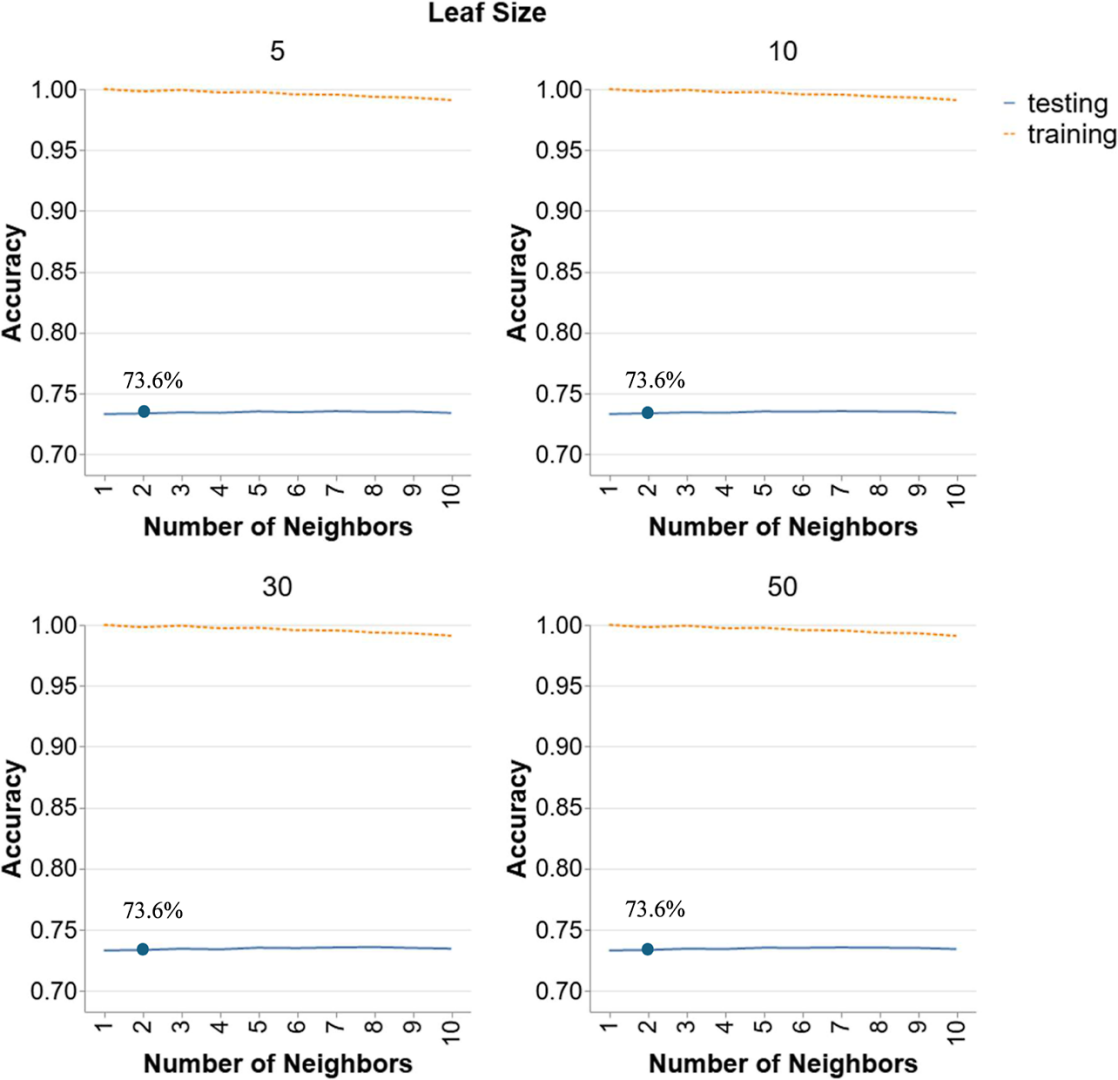
Hyperparameter tuning for *k*-NN models in Scenario 3.

#### 3.2.10 Scenario 4: RF

In Scenario 4, the model is trained on temperatures of 25, 300, 350, and 380 °C, and tested on the higher temperatures of 400 and 420 °C. This training configuration comprises only 42.8% of the data, meaning that the testing set makes up more than half of the data set. The temperature of 400 °C alone accounts for nearly half of the total data. Unlike previous scenarios, where training and testing were more balanced, Scenario 4 focuses on predicting higher temperature data from lower temperature data. The objective is to assess whether lower temperature data can be leveraged to predict higher temperature data, thus minimizing the need for extensive experimentation at elevated temperatures. Figure 10 demonstrates the performance of RF models in Scenario 4. Each graph corresponds to a different number of estimators (50, 100, 200, 300) and depths between 5 to 17. Testing accuracy starts at around 70% accuracy and increases gradually with depth, peaking around a maximum depth of 10 for all numbers of estimators. The highest testing accuracy is observed with a maximum depth of 10, achieving approximately 80% accuracy with 50 estimators. Beyond this point, testing accuracy stabilizes and shows minimal improvement, suggesting that additional depth does not enhance the model’s performance on unseen data. Increasing the number of estimators from 100 to 300 results in slightly better performance, but the overall impact is negligible. Consequently, the optimal hyperparameter combination for this model is with 50 estimators and a depth of 10 achieving an accuracy of 79.82%.

**Figure 10:**
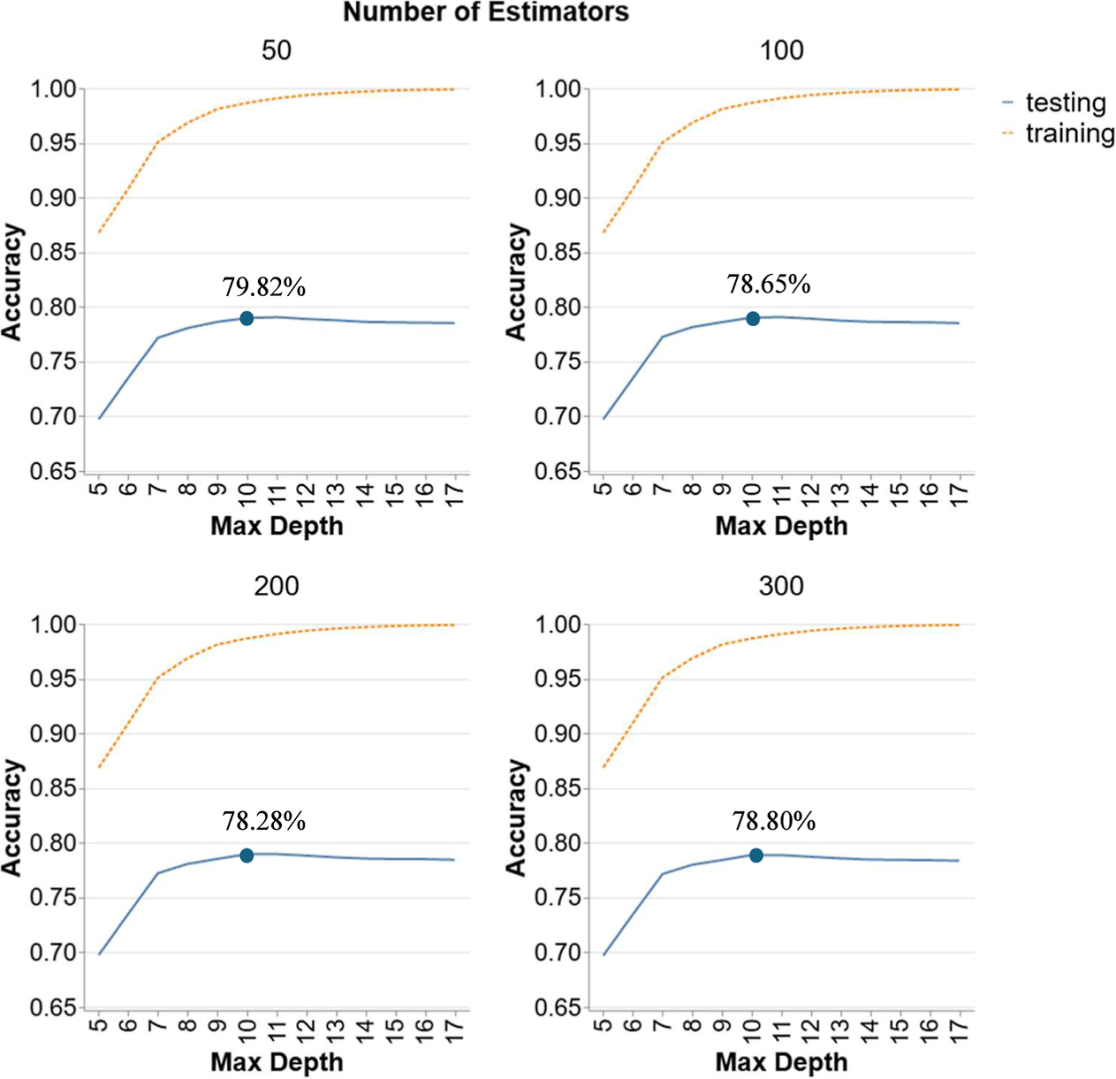
Hyperparameter tuning for RF models in Scenario 4.

#### 3.2.11 Scenario 4: GBR

Figure 11 showcases the performance of GBR models in Scenario 4. Each graph corresponds to a different number of estimators (50, 100, 200, 300) and compares two learning rates (0.1 and 1). For models with a learning rate of 0.1, testing accuracy starts around 78% and shows minimal improvement with increasing depth, peaking at approximately 78% to 79% across different depths and numbers of estimators. This suggests that a lower learning rate provides stable but modest improvements in testing accuracy. In contrast, models with a learning rate of 1 exhibit higher testing accuracy, starting around 80%. This higher learning rate allows the models to capture more complexity, resulting in better performance on unseen data. The testing accuracy remains relatively stable across different depths and numbers of estimators, indicating that while the higher learning rate improves performance, additional depth and more estimators provide declining outcomes. Increasing the number of estimators from 100 to 350 does not significantly impact the overall testing accuracy, suggesting that the chosen learning rate and depth are more critical factors in achieving optimal performance. Thus, the highest testing accuracy observed for this model is around 80.56%, achieved with 50 estimators and an LR of 1 at a depth of 20.

**Figure 11:**
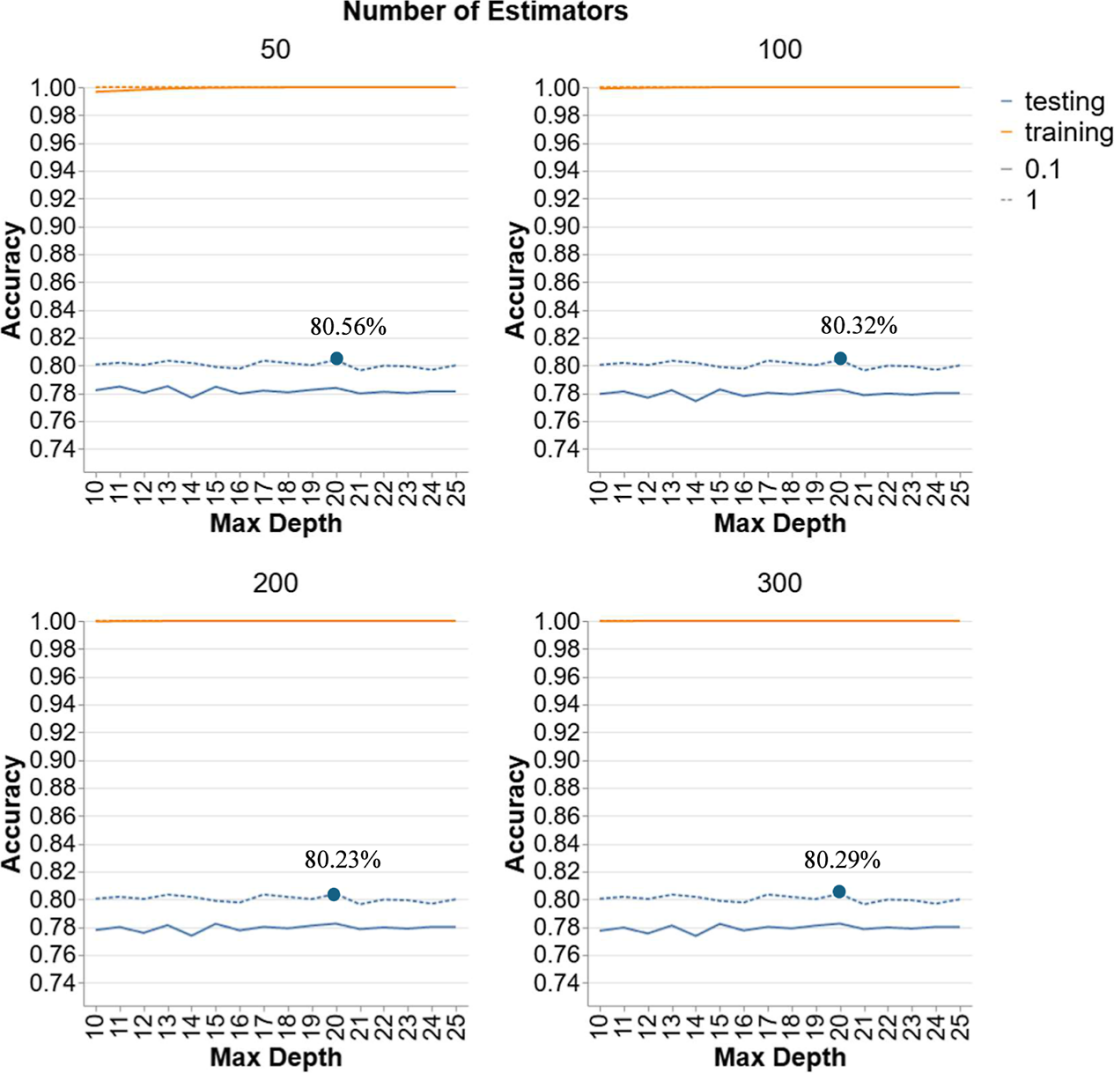
Hyperparameter tuning for GBR models in Scenario 4.

#### 3.2.12 Scenario 4: *k*-NN

Figure 12 illustrates the performance of *k*-NN models in Scenario 4. Each graph corresponds to a different leaf size (5, 10, 30, 50) with number of neighbors from 2 to 14. Testing accuracy shows minimal variation across different numbers of neighbors and leaf sizes. Testing accuracy remains relatively stable, hovering around 75%, and does not show significant improvement or decline as the number of neighbors increases from 2 to 14. As the number of neighbors increases, testing accuracy remains stable or slightly decreases, indicating that adding more neighbors does not enhance the model’s ability to generalize to unseen data in this scenario. The leaf size appears to have a negligible impact on testing accuracy, as the performance curves are nearly identical across all examined parameters. The maximum testing accuracy observed is approximately 79.67%, achieved with numbers of neighbors of 7 and leaf size of 5.

**Figure 12:**
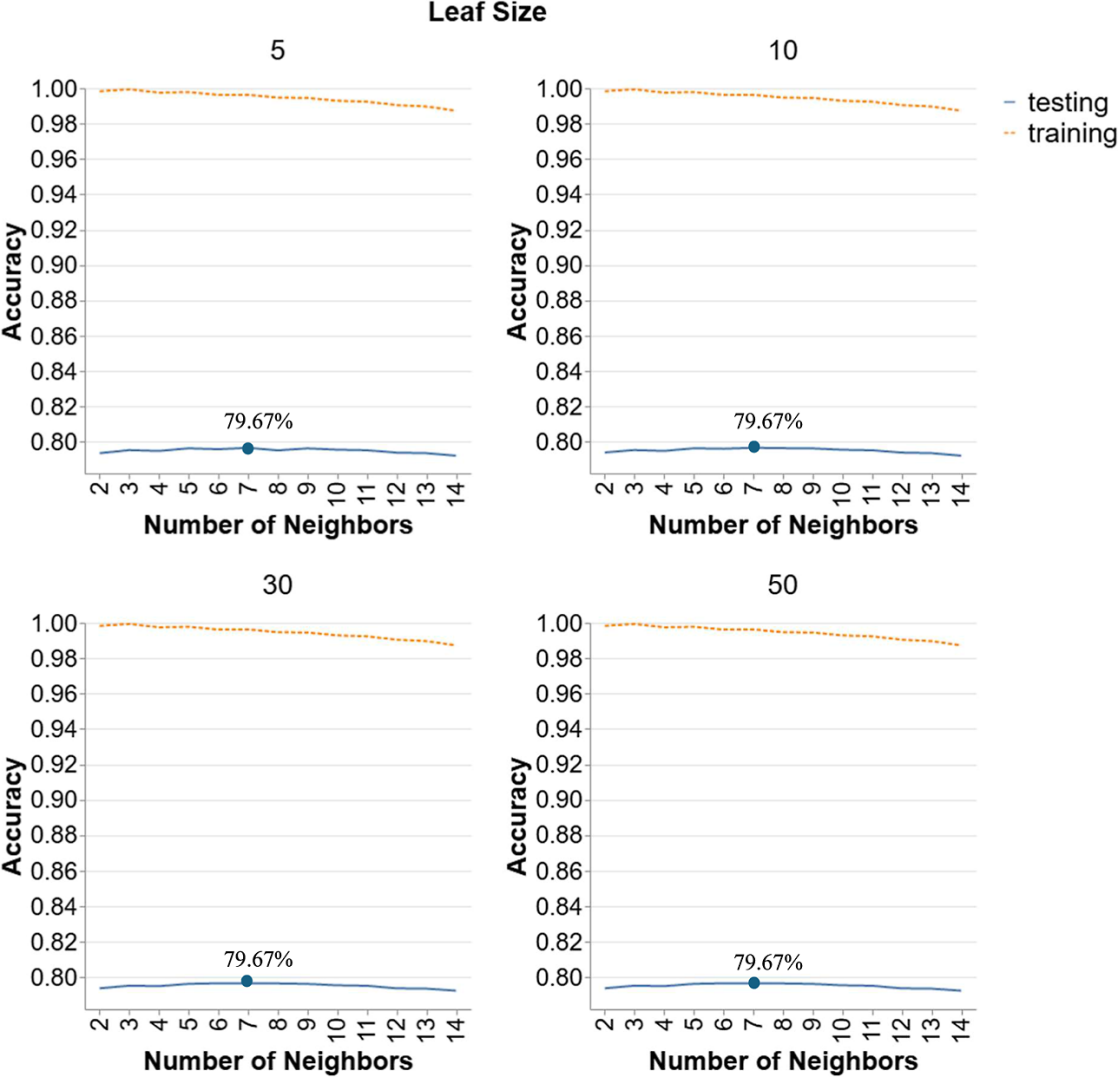
Hyperparameter tuning for *k*-NN models in Scenario 4.

### 3.3 Best-Performing Models from Hyperparameter Tuning

#### 3.3.1 Graphical Method (Visual)

Table 4 summarizes the results of the previous analysis through graphical hyperparameter tuning of the best-performing ML models across the four different scenarios. The goal of this analysis is to determine which model is most effective in each scenario, based on the best test accuracy, RMSE values, and the overfitting trend. The performance metrics are critical in selecting the best model for each scenario, aiming to minimize overfitting, reduce RMSE, and achieve the highest possible testing accuracy.

**Table 4:** All Scenarios: Best-Performing Models (Hyperparameter Tuning)

model	hyperparameters			test accuracy	train accuracy	overfit trend	train RMSE	test RMSE
Scenario 1								
RF	*D* = 20	*N* = 100		0.9936	0.9991	0.0055	0.1091	0.2913
GBR	*D* = 9	*N* = 300	LR = 0.2	0.9963	0.9997	0.0034	0.066	0.2119
*k*-NN	*N* = 2	LS = 5		0.9958	0.9979	0.0021	0.166	0.2350
Scenario 2								
RF	*D* = 8	*N* = 300		0.9437	0.9533	0.0096	0.8140	0.8052
GBR	*D* = 8	*N* = 50	LR = 0.1	0.9422	0.9925	0.0503	0.3260	0.8159
*k*-NN	*N* = 7	LS = 5		0.9196	0.9959	0.076	0.2398	0.9623
Scenario 3								
RF	*D* = 15	*N* = 150		0.7527	0.9963	0.2436	0.2202	1.839
GBR	*D* = 2	*N* = 200	LR = 0.5	0.9190	0.9683	0.049	0.6416	1.055
*k*-NN	*N* = 2	LS = 5		0.7360	0.9981	0.2621	0.1578	1.907
Scenario 4								
RF	*D* = 10	*N* = 50		0.7982	0.9868	0.1886	0.4386	1.568
GBR	*D* = 20	*N* = 50	LR = 1	0.8056	0.9912	0.1856	0.3476	1.436
*k*-NN	*N* = 7	LS = 5		0.7967	0.9963	0.1996	0.2310	1.574

In Scenario 1, GBR stands out with an astounding test accuracy of 99.63% and a train accuracy of 99.97%, showing minimal overfitting (0.0034). Both the train RMSE (0.066) and test RMSE (0.2119) are low, indicating that GBR achieves excellent generalization and prediction accuracy. Although the RF model also performs very well with a test accuracy of 99.36%, GBR’s slightly better performance in both RMSE and overfitting makes it the better choice in this scenario. In Scenario 2, the RF model is the best performer with a test accuracy of 94.37% and a train accuracy of 95.33%. While its test RMSE (0.8052) is higher than those in Scenario 1, the low overfit trend of 0.0096 indicates that RF is effectively generalizing without significant overfitting. The GBR model follows closely with a test accuracy of 94.22%, but its higher overfit trend (0.0503) makes it less optimal for this scenario compared to RF.

For Scenario 3, GBR model again emerges as the best model, with a test accuracy of 91.90% and a train accuracy of 96.83%. Although the overfitting is present (0.049), GBR’s test accuracy outperforms both RF (75.27%) and *k*-NN (73.60%) by a wide margin. The RMSE values of GBR, with a train RMSE of 0.6416 and a test RMSE of 1.055, suggest moderate errors, but GBR still provides the best balance of performance metrics in this scenario. In Scenario 4, GBR model again proves to be the top performer, with a test accuracy of 80.56% indicating solid predictive performance despite some overfitting. The RF model also performs well, with a test accuracy of 79.82%, but GBR’s higher accuracy and more reliable generalization make it the better option in this scenario.

Overall, the results clearly show that ensemble models (GBR and RF) perform the best across all scenarios. These ensemble methods are particularly well-suited for this data set because they combine the predictions of multiple individual models, leading to improved generalization and robustness against overfitting. The ability of ensemble methods to aggregate results from various decision trees (in RF) or boosting iterations (in GBR) helps them capture complex patterns and nuances in the data, which is likely why they outperformed *k*-NN, a simpler model that struggles with more complex data sets and other ML techniques that were previously explored including LinR, PLSR and SVR. Ensemble methods tend to be more effective in handling the variability in the data, leading to stronger overall performance across the board, as shown in previous works involving predictive modeling of characterization data from chemical processes as well.^16,55,56^

#### 3.3.2 Bayesian Optimization Method

The previous analysis involved manual prediction by performing individual model training for each set of ML techniques and hyperparameters through graphical demonstration. This labor-intensive and time-consuming process may have overlooked potentially other optimal hyperparameter combinations due to its inherent limitations in scope and efficiency. To enhance the accuracy and robustness of the model, automated hyperparameter tuning utilizing Bayesian Optimization is employed. Table 5 presents the outcomes of using Bayesian optimization to identify the best hyperparameters for different ML techniques across four scenarios in predicting FTIR intensities.

**Table 5:** Bayesian Optimization for all Scenarios

model	hyperparameters			test accuracyt	train accuracy	overfit trend	train RMSE	test RMSE
Scenario 1								
RF	*D* = 20	*N* = 100		0.9936	0.9991	0.0055	0.1091	0.2913
GBR	*D* = 10	*N* = 268	LR = 0.24	0.9965	0.9998	0.0033	0.046	0.2133
*k*-NN	*N* = 2	LS = 5		0.9958	0.9979	0.0021	0.166	0.2350
Scenario 2								
RF	*D* = 8	*N* = 300		0.9437	0.9533	0.0096	0.8140	0.8052
GBR	*D* = 8	*N* = 50	LR = 0.1	0.9422	0.9925	0.0503	0.3260	0.8159
*k*-NN	*N* = 7	LS = 24		0.9196	0.9959	0.076	0.2398	0.9623
Scenario 3								
RF	*D* = 15	*N* = 150		0.7527	0.9963	0.2436	0.2202	1.839
GBR	*D* = 2	*N* = 392	LR = 0.17	0.9215	0.9658	0.0443	0.6656	1.037
*k*-NN	*N* = 2	LS = 5		0.7360	0.9981	0.2621	0.1578	1.907
Scenario 4								
RF	*D* = 10	*N* = 50		0.7982	0.9868	0.1886	0.4386	1.568
GBR	*D* = 20	*N* = 50	LR = 1	0.8056	0.9912	0.1856	0.3476	1.436
*k*-NN	*N* = 7	LS = 5		0.7967	0.9963	0.1996	0.2310	1.574

The results from Table 5, which used Bayesian optimization to fine-tune hyperparameters, corroborate the findings from Table 4, which focused on graphical hyperparameter tuning. For RF and *k*-NN, the hyperparameter configurations obtained through Bayesian optimization provided the same results as those achieved through graphical tuning. This suggests that the hyperparameter combinations explored in the graphical tuning were already effective and that Bayesian optimization did not uncover better configurations for these models. However, for GBR, the results were different. In Scenario 2 and Scenario 4, Bayesian optimization found the same optimal hyperparameters as those identified through graphical tuning, yielding identical performance metrics. In contrast, in Scenario 1, Bayesian optimization improved the test accuracy slightly from 99.63% (obtained via graphical tuning) to 99.65%.

In Scenario 3, it increased the test accuracy from 91.90% (graphical tuning) to 92.15%. These improvements highlight the effectiveness of Bayesian optimization in identifying superior hyperparameter settings, particularly in scenarios where the graphical tuning might not have explored the optimal regions of the hyperparameter space. Overall, while Bayesian optimization reaffirmed the results for RF and *k*-NN, it demonstrated the ability to enhance the performance of GBR, suggesting its potential for further optimizing model accuracy in certain scenarios. The final selected model of each scenario was chosen based on the highest accuracy, lowest RMSE and lowest overfit trend as presented in Table 6.

**Table 6:** Final Selected Model for Each Scenario

model	hyperparameters			test accuracy	train accuracy	overfit trend	train RMSE	test RMSE
Scenario 1								
GBR	*D* = 10	*N* = 268	LR = 0.24	0.9965	0.9998	0.0033	0.046	0.2133
Scenario 2								
RF	*D* = 8	*N* = 300		0.9437	0.9533	0.0096	0.8140	0.8052
Scenario 3								
GBR	*D* = 2	*N* = 392	LR = 0.17	0.9215	0.9658	0.0443	0.6656	1.037
Scenario 4								
GBR	*D* = 20	*N* = 50	LR = 1	0.8056	0.9912	0.1856	0.3476	1.436

Thus, with the final ML models for each scenario having been selected as shown in Table 6 through Bayesian Optimization, several hidden trends emerge, revealing deeper insights into the performance differences across scenarios. Notably, scenarios 1–3 show better performance (above 92% accuracy), likely due to the inclusion of data from 400 °C in the training sets, which constitutes 45.7% of all data points. This substantial representation ensures that the models trained in these scenarios are exposed to the majority of the data points, enhancing their ability to generalize. Conversely, Scenario 4 tests on 400 °C without training on this critical temperature, leading to less accurate performance (maximum accuracy of 80.4%) due to the models’ unfamiliarity with this significant portion of the data. However, 80.4% is considered a good result, especially considering that only 42.8% of the data is being trained on while more than half is being tested, it demonstrates the robustness of the GBR model and its ability to generalize well, even with such a limited training set. Consequently, another noticeable trend is the stellar performance of GBR models across all scenarios. This is likely due to GBR’s ability to sequentially correct errors, leveraging the strengths of gradient boosting to enhance predictive accuracy.

Additionally, GBR’s ensemble nature, combining multiple weak learners, provides a robust framework for capturing complex patterns, whereas RF’s variability in performance suggests sensitivity to hyperparameter tuning, with deeper trees potentially leading to overfitting. *k*-NN’s reliance on nearby data points makes it less effective in scenarios with significant variability, as it directly depends on the proximity of data points, which may not capture the underlying global trends as effectively as GBR. In addition, lower depths resulted in higher accuracies in GBR models which can be attributed to the method’s iterative approach, where shallow trees prevent overfitting by maintaining a focus on broad trends rather than noise. Moderate learning rates ensures that each boosting step makes a meaningful contribution to reducing error without overcorrecting, unlike higher learning rates that can cause the model to overfit quickly by making larger updates.

Figure 13 shows a visualization of the results obtained from the Bayesian optimization process presented in Table 5.

**Figure 13:**
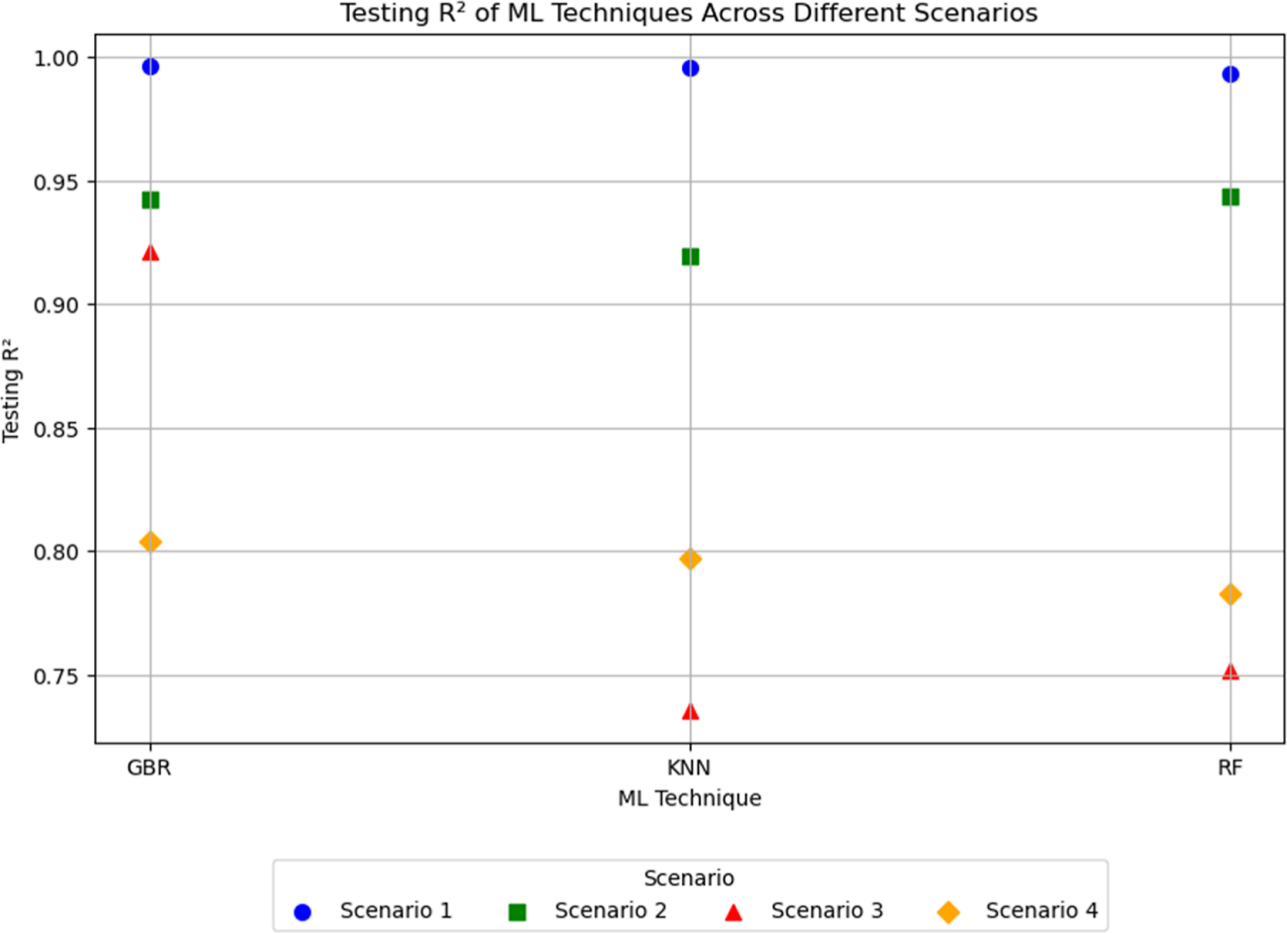
Prediction accuracy of the 3 ML Techniques across all scenarios.

Upon further inspection of Figure 13, the scatter plot reveals a significant anomaly in Scenario 3, where the performance of the ML techniques diverges more noticeably compared to the other scenarios. In Scenarios 1, 2, and 4, the testing *R* ^2^ values for GBR, *k*-NN, and RF are relatively close, indicating consistent performance across different techniques. However, in Scenario 3, there is a marked drop in accuracy for RF and *k*-NN, while GBR maintains a higher level of performance. This suggests that Scenario 3 presents unique challenges not present in the other scenarios. In Scenario 3, the training data includes temperatures of 350 °C, 380 °C, and 400 °C, while the testing data spans a broad range of 25 °C, 300 °C, 320 °C, and 420 °C. This high diversity between training and testing conditions makes it difficult for RF and *k*-NN to generalize effectively. The significant drop in RF’s testing *R* ^2^ value indicates that its ensemble method, which relies on aggregating multiple decision trees, struggles with the specific characteristics or complexity of the data in this scenario. Conversely, GBR’s iterative boosting approach seems more resilient to these challenges, enabling it to maintain better performance despite the constraints.

Figure 14 shows a 3D plot of GBR model predictions for Scenario 1 which is the most accurate model obtained from this analysis.

**Figure 14:**
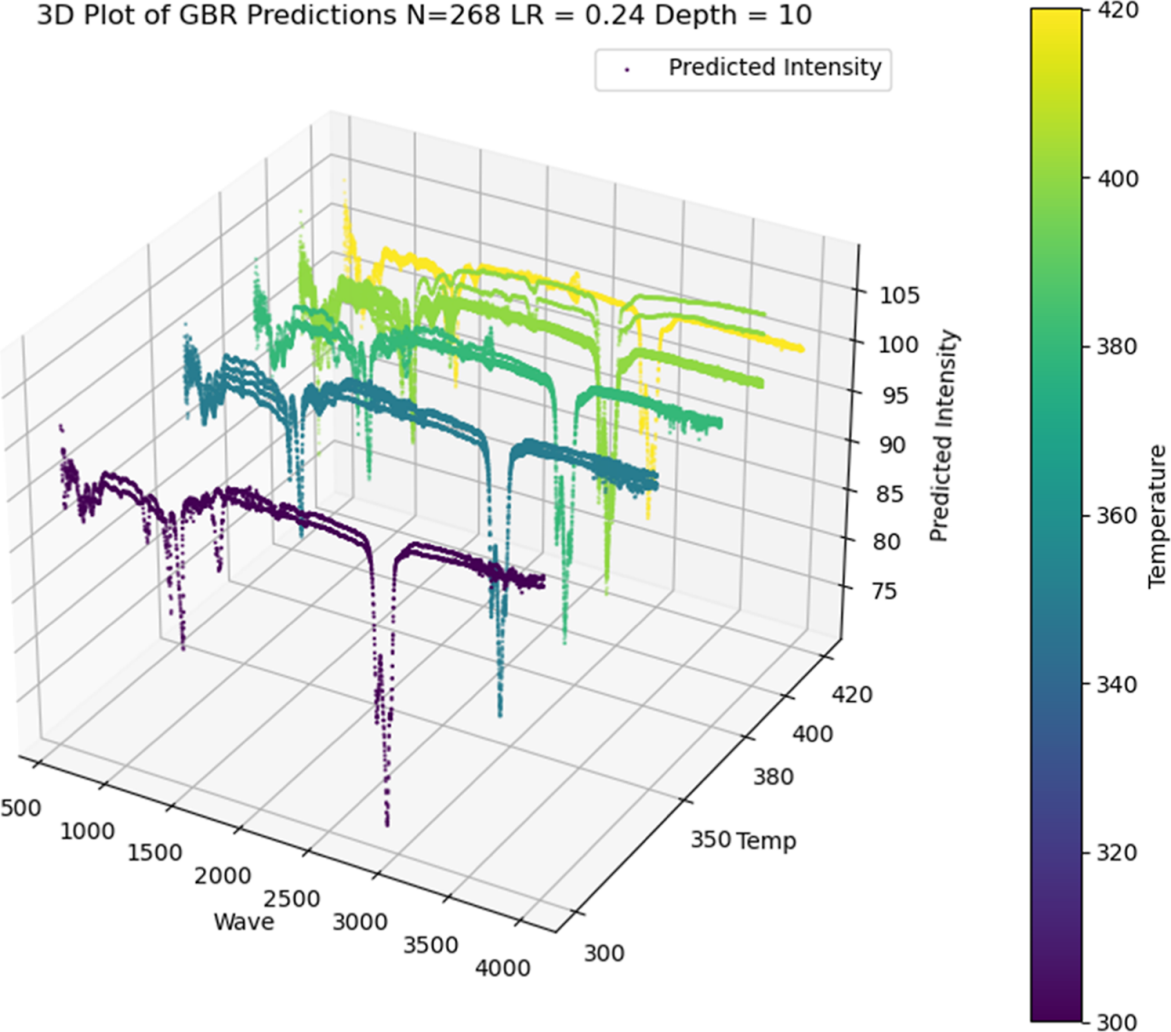
3D Plot of GBR Intensity for predictions in Scenario 1.

## 4 Conclusions

The analysis of predictive modeling using ML techniques across four different scenarios for predicting FTIR intensities reveals important insights into their performance, generalization capabilities, and adaptability to varying conditions. Scenario 1, using an 80/20 train-test split on all data points with 10-fold CV, showed that ensemble methods like RF and GBR excel in performance, achieving high accuracies and low RMSE values. Scenario 2, with training on temperatures of 25, 350, and 400 °C and testing on 300, 380, and 420 °C, introduced the challenge of temperature variation. In this scenario, RF and GBR models still performed well, but the differences between training and testing accuracies highlighted the importance of robust model tuning to handle such variability. Scenario 3, which trained on temperatures of 350, 380, and 400 °C and tested on 25, 300, and 420 °C, further emphasized the robustness of ensemble methods. While RF models exhibited good performance, GBR models, particularly those with higher learning rates and shallower depths, demonstrated superior generalization, achieving high testing accuracies and lower RMSE values. Scenario 4, with training on temperatures of 25, 300, 350, and 380 °C and testing on 400 and 420 °C, reinforced these findings. LinR and PLSR consistently showed poor performance across all scenarios, indicating their inability to capture complex patterns and adapt to new conditions. Whereas SVR was too computationally expensive to investigate further, taking around 7 to 8 h for a single reading. In contrast, GBR models, particularly with high learning rates and optimal depths, consistently provided the best balance of training and testing accuracy, low RMSE, and reasonable overfitting tendency. Across all scenarios, RF and GBR models stood out for their ability to handle complexity and variability, though careful hyperparameter tuning was essential to avoid overfitting and ensure robust generalization. *k*-NN models also performed well, especially in scenarios involving significant temperature variability, indicating their potential for applications where instance-based learning is beneficial. Hyperparameter tuning is crucial for these ensemble methods to achieve optimal performance and generalization, ensuring robust and accurate predictions across diverse temperature ranges. Thus, Bayesian optimization revealed the superiority of ensemble methods, particularly GBR, in managing the complexities and variabilities inherent in FTIR intensity prediction. These methods’ ability to iteratively refine predictions and aggregate multiple weak learners ensures robust performance across different conditions. The findings also emphasize the significant role of representative training data that encompasses the full range of expected testing conditions to ensure accurate and reliable predictions.

In summary, Bayesian optimization corroborated the findings from the graphical hyperparameter tuning and provided optimal hyperparameter configurations that improved performance. This finding demonstrated that with meticulous hyperparameter tuning, GBR and RF models can achieve exceptional performance in predicting FTIR intensities for complex feedstocks and characterization data from their thermal conversions, outperforming *k*-NN, SVR, linear regression and PLSR methods. The adaptability and robustness of these models make them highly suitable for applications involving complex and variable data, reinforcing the value of ensemble techniques in predictive modeling tasks.

These regression models can readily be applied for predicting process data from characterization of other compositionally complex feedstocks and their chemical conversions/reactions/processing such as conventional and halophytic biomass, proteins, polymer composites that are used in a variety of applications, soft-matter electronics, compositions of cancer inhibitors and other high-molecular weight molecules with dynamic physicochemical properties as well as solid–liquid feedstock such as sludge, industrial slurries and their processing at various stages. The ability to build soft-sensors having direct applications in predicting compositions, nonlinear laboratory and industrial characterization data, as well as establishing nonlinear spectrum-property-composition relationships for a variety of feedstock using decision-tree based ML models as the building blocks is a major future application of our work.

## Supplementary Material



## Notes

Brief theory of *k*-nearest neighbors (*k*-NN) and support-vector regression (SVR) are provided in the Supporting Information document for this manuscript (PDF)
