# Supplementary material for: Machine Learning-Based Predictive Modeling of Infrared Spectroscopic Data from Thermal Conversion of Athabasca Bitumen
Source: ACS Omega. 2025 Jul 2;10(27):29836–55. doi: 10.1021/acsomega.5c04463 (PMC12268744; doi:10.1021/acsomega.5c04463)
Supplement: Supplementary file 1 [file ao5c04463_si_001.pdf]

Supplementary Materials

Machine Learning-based Predictive Modeling of Infrared Spectroscopic data from Thermal Conversion of Athabasca Bitumen

Noora Al Mansoori[+], Munawar Abdul Shaik[+], Kaushik Sivaramakrishnan*[+]
[+]UAE University, Department of Chemical and Petroleum Engineering, Khalifa Street 15551, Al Ain, United Arab Emirates (UAE)
*Corresponding author email: kausiva@uaeu.ac.ae

***k*-nearest neighbors (*k*-NN)**

k-NN algorithm is a non-parametric, instance-based learning method used for regression tasks, where the goal is to predict continuous target values based on the characteristics of nearby data points in the feature space. Unlike parametric models that assume a specific functional form for the data, *k*-NN relies solely on the relationships among data points, making it particularly effective in capturing complex patterns when sufficient data is available. The fundamental principle of *k*-NN regression is that the target value of a query point is determined by aggregating the target values of its $\boldsymbol{k}$ nearest neighbours, as determined by a chosen distance metric. [1] In regression, the algorithm starts by computing the distances between the query point and all data points in the training set. These distances are typically measured using distance metrics in Cartesian coordinates such as the Euclidean or Pythagorean distance, defined mathematically as: [2]

$$d\,(x_1, x_2) = \sqrt{\sum_{j=1}^{d} (x_{1j} - x_{2j})^2} \tag{S1}$$

where $\boldsymbol{x_1}$ and $\boldsymbol{x_2}$ are two points in a $\boldsymbol{d}$-dimensional space. Once the distances are computed, the algorithm identifies the *k*-nearest neighbours, which are the data points with the smallest distances to the query point. The predicted value for the query point is then obtained by averaging the target values of these *k* neighbors. The prediction $\hat{\boldsymbol{y}}$ for a query point $\boldsymbol{x}$ is expressed as: [3]

$$\hat{y} = \frac{1}{k} \sum_{i=1}^{k} y_i \tag{S2}$$

where $\boldsymbol{y_i}$ represents the target value to the $\boldsymbol{i^{th}}$ nearest neighbour. The performance of *k*-NN regression is influenced by several hyperparameters, among which *n_neighbors* (the number of neighbors, *k*) and the *leaf_size* (LS) play pivotal roles. [4] The hyperparameter *n_neighbors* determines the number of data points used to compute the prediction. A small *k* may result in high sensitivity to noise and underfitting, as predictions will depend on very few data points, potentially unrepresentative of the underlying pattern. Conversely, a large *k* smoothens predictions by incorporating more data points, which may lead to overfitting if local variations in the data are essential. Therefore, selecting an optimal *k* typically involves cross-validation to balance bias and variance in the model. The LS parameter impacts the efficiency of neighbour searches. It determines the size of the leaf nodes in the tree, with smaller values leading to deeper trees and potentially more precise distance calculations at the cost of increased computation time. Larger

leaf sizes reduce the depth of the tree, resulting in faster results but possibly less accurate neighbour selection. While LS does not directly influence prediction accuracy, it significantly affects computational performance, especially for large datasets with high dimensionality. [5]

Support Vector Regression (SVR)

SVR extends the SVM (Support Vector Machine) framework to regression tasks by predicting continuous outcomes while incorporating a margin of tolerance for errors. SVR finds a function that models the relationship between input features and the target variable while ensuring deviations remain within a specified margin, denoted as $\boldsymbol{\varepsilon}$. This margin, known as the $\boldsymbol{\varepsilon}$-insensitive tube, ignores small errors in the optimization process. [6] Only a subset of data points, called support vectors, influence the regression function by either lying on the boundary of the $\boldsymbol{\varepsilon}$-tube or exceeding it. Those exceeding the margin introduce slack variables, which quantify deviations and are penalized in the optimization process. The number of support vectors depends on $\boldsymbol{\varepsilon}$, where a larger $\boldsymbol{\varepsilon}$ reduces their count and a smaller $\boldsymbol{\varepsilon}$ increases sensitivity to variations. [7] The algorithm is particularly well-suited for datasets with nonlinear relationships due to its ability to employ kernel methods.

For the simplest kernel (linear), SVR seeks to identify a linear function expressed as:

$$f(x) = x^T\beta + \beta_0 \tag{S3}$$

where $\boldsymbol{x^T}$ is the feature vector and $\boldsymbol{\beta}$ is the coefficient vector, and $\boldsymbol{\beta_0}$ is the bias term that adjusts the output. The optimization objective minimizes model complexity while penalizing deviations beyond $\boldsymbol{\varepsilon}$, expressed as: [8]

$$min\left(\frac{1}{2}\,||\beta||^2 + C\sum_{i=1}^{N}(\xi_i + \xi_i{}^*)\right) \tag{S4}$$

where $\frac{\mathbf{1}}{\mathbf{2}}\,||\boldsymbol{\beta}||^{\mathbf{2}}$ ensures model simplicity, and $\boldsymbol{C}$ controls the trade-off between complexity and error penalties. The slack variables $\boldsymbol{\xi_i}$ and $\boldsymbol{\xi_i}^*$ measure underestimation and overestimation, respectively, beyond $\boldsymbol{\varepsilon}$, ensuring flexibility in handling outliers. [9]

SVR extends to nonlinear regression using kernel functions that transform input data into a higher-dimensional feature space. For more complex patterns, the polynomial kernel introduces nonlinearity by raising the dot product of input features to a specified degree $\boldsymbol{d}$, scaled by $\boldsymbol{\gamma_{Poly}}$, the polynomial scaling factor, and offset by a constant $\boldsymbol{r}$: [10]

$$K(x_i, x_j) = (\gamma_{Poly}\, x_i{}^T x_j + r)^d \tag{S5}$$

The radial basis function (RBF) kernel is highly flexible and can model intricate relationships by focusing on the similarity between data points. It is defined as: [11]

$$K(x_i, x_j) = \exp\left(-\gamma_{RBF}||x_i - x_j||^2\right) \tag{S6}$$

where γ_{RBF} controls the influence of individual data points. A higher γ_{RBF} value makes the kernel focus on points that are closer together, resulting in a highly localized model, while a lower γ_{RBF} value results in a smoother and more generalized model. Finally, the sigmoid kernel, inspired by neural networks, introduces nonlinearity through the hyperbolic tangent function. It is expressed as: [12]

$$K(x_i, x_j) = \tanh\left(\gamma_{Sig}\, x_i^T x_j + \theta\right) \tag{S7}$$

where $\boldsymbol{\gamma_{Sig}}$ is the sigmoid scaling factor that scales the dot product of the feature vectors and $\boldsymbol{\theta}$ shifts the activation threshold.

The hyperparameters $\boldsymbol{\varepsilon}$ and $\boldsymbol{C}$ which are used for all kernels, are critical in determining the performance of the SVR model. Additionally, $\boldsymbol{\gamma}$ is a hyperparameter specific to nonlinear kernels that controls how the feature space is mapped, affecting the model's ability to capture complex relationships. [12] Proper tuning of these hyperparameters, along with selecting the appropriate kernel and its associated parameters, ensures that the SVR model achieves a balance between capturing the underlying patterns in the data and generalizing well to unseen examples. Through this combination of regularization, tolerance, and flexibility offered by kernels, SVR is a powerful tool for regression tasks.
